# Comparative Genomics of *Acinetobacter baumannii* Clinical Strains From Brazil Reveals Polyclonal Dissemination and Selective Exchange of Mobile Genetic Elements Associated With Resistance Genes

**DOI:** 10.3389/fmicb.2020.01176

**Published:** 2020-06-17

**Authors:** Nilma C. Leal, Túlio L. Campos, Antonio M. Rezende, Cássia Docena, Carina L. Mendes-Marques, Felipe L. de Sá Cavalcanti, Gabriel L. Wallau, Igor V. Rocha, Carmelita L. B. Cavalcanti, Dyana L. Veras, Lilian R. Alves, Mariana Andrade-Figueiredo, Maria P. Silva de Barros, Alzira M. Paiva de Almeida, Marcia M. Camargo de Morais, Tereza C. Leal-Balbino, Danilo E. Xavier, Osvaldo P. de-Melo-Neto

**Affiliations:** ^1^Aggeu Magalhães Institute (IAM), Fundação Oswaldo Cruz (Fiocruz), Recife, Brazil; ^2^Department of Pathology, Institute of Biological Sciences, University of Pernambuco, Recife, Brazil; ^3^Laboratory of Immunopathology Keizo Asami, Federal University of Pernambuco, Recife, Brazil; ^4^Department of Tropical Medicine, Federal University of Pernambuco, Recife, Brazil

**Keywords:** *Acinetobacter baumannii*, antimicrobial resistance, virulence, mobile genetic elements, Brazil

## Abstract

*Acinetobacter baumannii* is an opportunistic bacterial pathogen infecting immunocompromised patients and has gained attention worldwide due to its increased antimicrobial resistance. Here, we report a comparative whole-genome sequencing and analysis coupled with an assessment of antibiotic resistance of 46 *Acinetobacter* strains (45 *A. baumannii* plus one *Acinetobacter nosocomialis*) originated from five hospitals from the city of Recife, Brazil, between 2010 and 2014. An average of 3,809 genes were identified per genome, although only 2,006 genes were single copy orthologs or core genes conserved across all sequenced strains, with an average of 42 new genes found per strain. We evaluated genetic distance through a phylogenetic analysis and MLST as well as the presence of antibiotic resistance genes, virulence markers and mobile genetic elements (MGE). The phylogenetic analysis recovered distinct monophyletic *A. baumannii* groups corresponding to five known (ST1, ST15, ST25, ST79, and ST113) and one novel ST (ST881, related to ST1). A large number of ST specific genes were found, with the ST79 strains having the largest number of genes in common that were missing from the other STs. Multiple genes associated with resistance to β-lactams, aminoglycosides and other antibiotics were found. Some of those were clearly mapped to defined MGEs and an analysis of those revealed known elements as well as a novel Tn*7*-Tn*3* transposon with a clear ST specific distribution. An association of selected resistance/virulence markers with specific STs was indeed observed, as well as the recent spread of the OXA-253 carbapenemase encoding gene. Virulence genes associated with the synthesis of the capsular antigens were noticeably more variable in the ST113 and ST79 strains. Indeed, several resistance and virulence genes were common to the ST79 and ST113 strains only, despite a greater genetic distance between them, suggesting common means of genetic exchange. Our comparative analysis reveals the spread of multiple STs and the genomic plasticity of *A. baumannii* from different hospitals in a single metropolitan area. It also highlights differences in the spread of resistance markers and other MGEs between the investigated STs, impacting on the monitoring and treatment of *Acinetobacter* in the ongoing and future outbreaks.

## Introduction

*Acinetobacter baumannii* is an opportunistic Gram-negative bacterium considered one of the most prevalent pathogens associated to nosocomial infections, especially among immunocompromised patients. Its ability to survive in hostile conditions, its high level of intrinsic and acquired antimicrobial resistance and the ease with which it spreads within and between health care units worldwide has made *A. baumannii* a successful pathogen in hospital settings (McConnell et al., 2013; Wong et al., 2017; Hamidian and Nigro, 2019). The genus *Acinetobacter* has also gained attention due to the increasing number of reported multi-drug resistant (MDR) clinical strains. These represent a great challenge in terms of treatment of infections and the elimination of pathogenic *Acinetobacter* species. The genome plasticity of *A. baumannii* further enhances its ability to adapt and persist in hospital environments and has facilitated the emergence of global MDR strains (Imperi et al., 2011; McConnell et al., 2013; Elhosseiny and Attia, 2018; Rocha et al., 2018).

*Acinetobacter baumannii* exhibits intrinsic resistance to many classes of antimicrobial agents and is further capable of developing resistance to virtually all other classes of agents used in the clinical practices to treat Gram-negative infections (Esterly et al., 2011; Leclercq et al., 2013; Nowak and Paluchowska, 2016; Lee et al., 2017). Considering the β-lactam antimicrobials, specially the carbapenems, one of the main therapeutic options for infections caused by most Gram-negative pathogens and which have been regarded as one of the last line agents for the infection therapy, a rapid increase in carbapenem-resistant *A. baumannii* (CRAb) has been observed (Pogue et al., 2013; Piperaki et al., 2019). In *A. baumannii*, a major mechanism of carbapenem resistance is related to carbapenemase enzymes belonging to the Ambler A, B, and D classes of β-lactamases (Patel and Bonomo, 2013; Rahman et al., 2018). Carbapenem-hydrolyzing class D β-lactamases (CHDL) are the most common in *A. baumannii* strains and these are referred as OXA-type carbapenemases (Nowak and Paluchowska, 2016). Other mechanisms that have been related to decreased carbapenem-susceptibility in *A. baumannii* include: decreased permeability due to changes in porin expression, especially CarO and OprD-like; overexpression of efflux pumps; changes in penicillin-binding proteins; and overexpression of intrinsic *Acinetobacter*-derived cephalosporinases (Fernández-Cuenca et al., 2003; Hu et al., 2007; Catel-Ferreira et al., 2011; Jeon et al., 2014). In MDR strains more than one of these mechanisms can work synergically (Sen and Joshi, 2016).

The sequencing of several *A. baumannii* genomes revealed a wide repertoire of antimicrobial resistance genes, many of which associated with transposable elements and insertion sequences and which might be found in genomic islands (GIs), known as AbaR (Zhu et al., 2013; Liu et al., 2014; Bi et al., 2019). Several AbaR islands have been described which can vary in size and are dynamically reshaped mainly due to the activity of transposases, recombinases and integrases (Krizova et al., 2011; Li et al., 2015; Hamidian and Hall, 2018; Bi et al., 2019). Resistance genes can also be found within plasmids, which can be exchanged intra- and interspecies (Leungtongkam et al., 2018; Wibberg et al., 2018) and even by prophages (Wachino et al., 2019). In contrast to the better understood resistance genes, few virulence mechanisms and associated genes have been identified in *A. baumannii* involved in the establishment and progression of infection (Morris et al., 2019). Nevertheless, some determinants have been found related to *Acinetobacter* virulence and these include factors involved in biofilm formation, secretion systems, surface glycoconjugates and micronutrient acquisition systems (Penwell et al., 2012; Carruthers et al., 2013; Kenyon and Hall, 2013; Weber et al., 2017; Harding et al., 2018; Morris et al., 2019). Even though some studies have suggested that *Acinetobacter* elaborates a lipooligosaccharide (LOS) layer instead of lipopolysaccharides (LPS) on their cell surface, the diversity of the biosynthesized LOS core has also been implicated in its survival and virulence (Weber et al., 2016).

Considering the *A. baumannii* genetic diversity, their strains have been typed by the multilocus sequence typing method (MLST) to distinguish between different clonal lineages. Based on two existing MLST schemes, Pasteur (Diancourt et al., 2010) and Oxford (Bartual et al., 2005), clinical isolates of *A. baumannii* have been clustered into several clonal complexes (CCs) that group genetically and phenotypically related strains that are generally fairly widespread across the globe. More recently the Pasteur scheme has been shown to me more reliable and appropriate for evaluations concerning epidemiological studies (Gaiarsa et al., 2019). Eighteen of those clonal complexes were early on considered international clones since they were been found in more than one continent, while the remaining were suspected to be restricted to Europe and Asia (Karah et al., 2012). In Brazil, many multidrug resistant clinical strains of *A. baumannii* collected from several states have been studied (Pagano et al., 2017) and at least six clonal complexes were identified (Chagas et al., 2014). The CC79, CC1, CC15, and CC113, complexes (named according to the Pasteur scheme) have been more frequently reported among Brazilian clinical strains of *A. baumannii*, but only CC79 remains predominantly restricted to South America, while the others are considered international clones (Karah et al., 2012; Chagas et al., 2014; Girlich et al., 2014).

Although *Acinetobacter* MDR strains belonging to different clonal complexes have been reported from Brazil (Chagas et al., 2015; Turano et al., 2016; Pagano et al., 2017; Da Silva et al., 2018), no comprehensive genome-wide comparative analysis of Brazilian strains has been conducted and a need for further sequencing from South American strains has recently been pointed out (Hamidian and Nigro, 2019). In the present study, we sequenced the genomes of 46 *Acinetobacter* clinical strains from five different hospitals located in Recife, a large metropolitan area of Northeastern Brazil, to reveal the genetic features and the outbreak potential of multiple clonal lineages and circulating strains of this pathogen. An extensive genome-wide comparative analysis based on the sequenced genomes was carried out, leading to the identification of key genetic determinants associated with antimicrobial resistance, virulence and associated transposable elements.

## Materials and Methods

### Bacterial Isolates, Growth Conditions and Antimicrobial Susceptibility Testing

This study evaluated a total of 45 carbapenem-resistant *A. baumannii* (CRAb) strains and a single pan-susceptible clinical strain of *A. nosocomialis* (Acb_11) (Supplementary Table S1). These strains were collected between 2010 and 2014 from patients hospitalized at five different tertiary hospitals located in Recife, Brazil. A single *A. baumannii* strain per patient was included in this study and those were recovered from upper respiratory tract infections (*n* = 13); bloodstream (*n* = 10), cerebrospinal fluid (CSF, *n* = 09), peritoneal fluid (*n* = 03); catheter tip (*n* = 03); urinary tract (*n* = 02); bone (*n* = 01); soft tissues (*n* = 1), and rectal swab cultures (*n* = 04). The strains were stored at −80°C in commercial Brain-Heart Infusion (BHI) broth supplemented with 20% glycerol for preservation and were grown in BHI medium at 35 ± 2°C.

Species identification was verified by Biotyper MALDI-TOF mass spectrometry (Marí-Almirall et al., 2017). Clinical and Laboratory Standards Institute (CLSI) broth microdilution susceptibility testing was performed to determine the minimal inhibitory concentration (MIC) to ampicillin/sulbactam (1/0.5–128/64 μg/ml); ceftriaxone (1–128 μg/ml); ceftazidime (1–128 μg/ml); cefepime (1–128 μg/ml); imipenem (0.5–64 μg/ml); meropenem (0.5–64 μg/ml); ciprofloxacin (0.25–32 μg/ml); levofloxacin (0.25–32 μg/ml); amikacin (2–256 μg/ml); gentamicin (0.5–64 μg/ml), and polymyxin B sulfate (0.25–32 μg/ml). *Pseudomonas aeruginosa* ATCC 27853 and *Escherichia coli* ATCC 25922 strains were used as quality controls in this assay (Clinical and Laboratory Standards Institute [CLSI], 2015, 2018).

### Library Preparation and Sequencing

Total DNA from each strain was extracted employing the DNeasy^®^ Blood and Tissue kit (QIAGEN^®^, Hilden, Germany) following the manufacturer’s instructions. After DNA extraction, the DNA was quantified with the QUBIT fluorometric device (Thermo Fisher^®^, Waltham, MA, United States) using the Qubit dsDNA BR Assay Kit (Thermo Fisher^®^, Waltham, MA, United States). Adapter-ligated sequencing libraries were prepared using the Nextera XT DNA Library Prep Kit (Illumina, San Diego, CA, United States) with 1 ng input of genomic DNA for each sample. Oligonucleotides used as indexes were added by PCR amplification according to the manufacturer’s instructions. Unique index-tagged libraries were generated for each strain and pooled to generate a multiplexed library which was sequenced on Illumina MiSeq in a single run, using MiSeq Reagent Kit v3, 2 × 300 base pair run. The raw sequencing data generated in the present study is publicly available at the European Nucleotide Archive (ENA), accession: PRJEB12754^1^.

### Genome Assembly, Gene Prediction, and Annotation

Prior to genome assembly, the quality of the raw sequencing reads was first evaluated using the FastQC package^2^, followed by sequence trimming using the Trimmomatic v0.32 program (Bolger et al., 2014) with the following parameters: LEADING:3 TRAILING:3 SLIDINGWINDOW:4:15 MINLEN:50 CROP:240. For each strain, the trimmed paired-end data was then used to perform genomic *de novo* assembly with the VelvetOptimiser^3^ script for Velvet (Zerbino, 2010), setting N50 optimization function for *k*-mer length selection. For protein prediction and annotation the Prokka pipeline (Seemann, 2014) was used along with a database containing all *A. baumannii* proteins available at NCBI Refseq, to enhance this annotation (parameters: –genus and –gram neg).

In order to visualize genomic contexts, a synteny map was created using the Mauve software (Darling, 2004) to align all 46 sequenced genomes. The produced alignment was then converted to the ClustalW format (Thompson et al., 2003) and the GBrowse Syn framework (McKay et al., 2010) used to upload it to a relational database and to visualize the synteny maps in an Internet browser.

All draft assemblies and annotations produced in the present study are available at https://doi.org/10.6084/m9.figshare.12144855.

### Phylogenetic Tree and MLST Analysis

To predict the ortholog protein groups used in this study, the fourteen *A. baumannii* reference proteomes available at the time of the last collection of our samples were downloaded from NCBI and used as input for ORTHOMCL (Li et al., 2003) along with the Prokka-predicted proteomes of all the strains sequenced here. To generate a phylogenetic tree using an alignment-free approach, first we generated 1,000 datasets using a bootstrap approach (bootstrap sampling with replacement), picking 1740 ORTHOMCL groups with replacement (the number of orthologs identified for all the strains used in the analyses), using the *sample* function in R - http://www.R-project.org. Each ortholog group included one protein for each sample present in this analysis, and these groups were defined as the list of core proteins. For each bootstrap, amino-acid sequences for each ortholog group were extracted from the predicted proteome of each strain. Each pool of protein sequences in each bootstrap was then used as input for CVTree (Qi et al., 2004; Xu and Hao, 2009; Zuo and Hao, 2015), a software that generates phylogenies based on a composition vector approach and that uses alignment-free whole genome comparisons, yielding 1,000 distance matrices. The Neighbor program from the PHYLIP package (Retief, 2000) was then used to generate a phylogenetic tree for each distance matrix, with the Consense program, also from PHYLIP, used to compute a consensus tree by the majority-rule consensus tree method, followed by tree visualization and figure generation using the iTOL software (Letunic and Bork, 2016). The pan-susceptible *A. nosocomialis* Acb_11 clinical strain was used as an outgroup.

Multilocus sequence typing (MLST) analysis was performed using SRST2 (Inouye et al., 2014). The MLST profiles were assigned *in silico* using the marker sequences available at the *A. baumannii* MLST Databases, PubMLST^4^ for the Pasteur (Diancourt et al., 2010) scheme. Allele sequences were extracted from each *Acinetobacter* genome using the BLASTN tool and were then submitted to the pubMLST database for the assignment to both existing and new sequence types (STs).

NCBI accession numbers for the reference *A. baumannii* genomes included in the present study: AB0057 - GCA_000021245; AB307-0294 - GCA_000021145; AYE - GCA_000069245; BJAB0715 - GCA_000419405; D1279779 - GCA_000186665; ZW85-1 - GCA_000505685; BJAB0868 - GCA_000419425; BJAB07104 - GCA_000419385; MDR-ZJ06 - GCA_000226275; MDR-TJ - GCA_000187205; TYTH-1 - GCA_000302575; 1656-2 - GCA_000188215; ACICU - GCA_000018445; ATCC 17978 - GCA_000015425.

### Identification of Antibiotic Resistance and Virulence Genes and Features

In order to identify the resistance genes, we queried the predicted protein sequences against the ResFinder (Zankari et al., 2012) using *blastp* (*e*-value cutoff 1*e*-5) (Altschul et al., 1990), keeping only genes that displayed coverage higher than 90% and identity values above 70%. The *blaADC* alleles genes and their association with ISAba-like elements were confirmed through PCR and DNA sequencing, as previously described (Ruiz et al., 2007). To search for virulence related genes in the sequenced genomes, as well as in the reference *A. baumannii* genomes selected for this study, we carried out BLAST searches for genes classified within the virulence factor of pathogenic bacteria database (VFDB) for the *Acinetobacter* genus with the VFanalyzer online tool (Chen, 2004; Liu et al., 2019). Genes encoding the BfmRS two-component regulatory system (Geisinger and Isberg, 2015) were also investigated. A presence/absence table was generated based on the selected resistance and virulence related genes found. This table was used as input for iTOL (Letunic and Bork, 2016) for visualization alongside the phylogenetic tree.

### Analysis of Capsule (K) and O-Antigen (OC) Biosynthetic Gene Clusters

To characterize predicted protein sequences from the K and OC loci, a preliminary assessment was carried out using both *blastp* (Altschul et al., 1990) and Pfam (Bateman, 2002), for gene identification, and Artemis (Rutherford et al., 2000), to compare gene arrangement. Modules A and B from the K loci and the OC loci were first identified using *blastp* queries against the flanking *fkpA*/*lldP*, and *ilvE*/*aspS* genes, respectively, as described previously (Kenyon and Hall, 2013; Kenyon et al., 2014; Holt et al., 2016). Variable genes encoding proteins predicted to be involved in sugar synthesis were examined for homology to the *A. baumannii* reference sequences. KL and OCL types were subsequently defined using the *Kaptive* tool (Wick et al., 2018) and the curated databases of annotated reference sequences for *A. baumannii* K and OC loci, recently described (Wyres et al., 2020). For the gene encoding the WaaL O-antigen ligase, we searched for homologs to the *P. aeruginosa* PAO1 [GenPept accession NP_253686.1] and to WaaL ligases from *E. coli* core types K12, R1, R2, R3, and R4. Similar searches were carried out for the gene encoding the PglL *O*-oligosaccharyltransferase.

### Search for Genomic Islands and Mobile Genetic Elements (MGEs)

Nucleotide sequences encoding published genomic islands (GIs) were downloaded from PAIDB (Yoon et al., 2015). For the identification of GIs within our strains, two different approaches were then applied. First, a homology-based search method against known islands was followed by a second approach which evaluated the flanking sequences of known insertion hotspots. Fragments with high similarity to AbaR0 (KF483599) and AbGRI1^5^ were thus identified and aTRAM (Allen et al., 2015) was used to map the sequenced reads from identified resistance genes and virulence factors onto these GIs. Mapping coverage was also evaluated to identify the potentially most similar GIs present in each strain.

Nucleotide sequences annotated by the Prokka software that encode transposases were also recovered and used in an alignment search with the *blastp* tool (with default parameters) against the ISfinder database (Siguier, 2006). This was performed in order to identify the corresponding IS family of transposases. If no homolog could be detected in the ISfinder database, we then performed a complementary search against the NCBI non-redundant database. In order to access the copy number of each element *per* strain, we used the Prokka annotated transposases to perform *blastn* searches against contigs from all 46 strains and the 14 reference genomes.

Prophages derived sequences were detected using the PhiSpy software (Akhter et al., 2012) which uses an *ab initio* approach to find phage genome related segments.

## Results

### Strain Selection and Antimicrobial Susceptibility Profile

In order to investigate *Acinetobacter* clinical strains associated with hospital acquired human infection in Northeastern Brazil, we selected 46 *Acinetobacter* strains from patients hospitalized in five hospitals from Recife. Forty-five of those were carbapenem-resistant *A. baumannii* strains isolated from different sources or body fluids. A single pan-susceptible strain of *A. nosocomialis* (Acb_11), a related nosocomial species from the *Acinetobacter calcoaceticus-baumannii* complex, was isolated from a bloodstream infection. All strains were identified by MALDI-TOF (Marí-Almirall et al., 2017), with the species identification subsequently confirmed by the genome sequence analysis, as described below. The *A. nosocomialis* strain (Acb_11) was maintained in our analysis considering that it turned out to be an efficient outgroup species for the phylogenetic studies carried out.

Prior to any genetic analysis, we opted to determine the antimicrobial susceptibility profile exhibited by the 46 *Acinetobacter* clinical strains against eleven different antimicrobial agents using the CLSI broth microdilution method (results summarized in the Supplementary Table S1). As expected, the *A. nosocomialis* Acb_11 was the sole strain susceptible to all tested antimicrobial agents. In contrast, after testing with imipenem and meropenem, the carbapenem-resistance phenotype was confirmed for all 45 *A. baumannii* strains. Among these clinical strains, nearly all were also resistant to the first two cephalosporins tested, ceftriaxone and ceftazidime, with two thirds also found to be resistant to a third cephalosporin, cefepime. A reduced susceptibility to cefepime was, nevertheless, observed for most of the remaining strains (30%, 13/45), with only a single strain (Acb_41) being susceptible to the three cephalosporins tested.

The totality of the strains included in this study showed *in vitro* susceptibility to ampicillin/sulbactam. In contrast, distinct results were observed regarding resistance to the two aminoglycosides tested, amikacin and gentamicin. Full resistance to amikacin was found in 60% of the strains, with 20% classified as susceptible and the remaining 20% having an intermediate resistance phenotype, while for gentamicin a resistance phenotype was found for ∼50% of the strains, with ∼40% classified as susceptible. Cross-resistance was observed for the gentamicin resistant strains only, since those also had reduced susceptibility to amikacin, while eight of the amikacin-resistant strains were nevertheless susceptible to gentamicin. Regarding the fluoroquinolones tested, ciprofloxacin, and levofloxacin, all 45 strains were resistant to ciprofloxacin and even the highest concentration tested (32 μg/ml) was incapable of inhibiting the growth of most of the strains. In contrast, levofloxacin resistance was observed for only ∼13% of the strains. The last antimicrobial agent investigated was polymyxin B, which was found to be active against all tested *A. baumannii* strains. Overall, the antibiotic resistance profile from the selected *A. baumannii* strains confirm a diverse pattern of resistance to cephalosporins, fluoroquinolones, and aminoglycosides, while displaying full resistance to the two carbapenems tested and susceptibility to the ampicillin/sulbactam combination and to polymyxin B.

### *Acinetobacter* Genomic Features

To evaluate the genetic diversity of the clinical strains selected for this study as well as to define their phylogenetic relationships and understand the genetic basis for their diverse antibiotic resistance profiles, we performed next generation sequencing, draft genome assembly and annotation for all 46 strains. The assembly metrics and statistics for the sequenced genomes can be found in the Supplementary Table S2. We identified an average of 3,809 genes per genome but only 2,006 genes were single copy orthologs, or core genes that are conserved across all 46 *Acinetobacter* strains sequenced, similar to results derived from other analysis (Imperi et al., 2011; Chan et al., 2015). The average number of accessory genes per genome was 2,106 highlighting the high degree of diversity in gene repertoire between different strains. This is better visualized through an ortholog group accumulation curve (shown in Supplementary Figure S1A) where the total number of ortholog groups identified are plotted according to the total number of strains sequenced. The curve shows a continuous increase in the number of ortholog groups that does not reach a plateau even after adding all 46 genomes sequenced, indicating that each new strain adds new genes that are not found in the previously added genomes. Indeed, an average number of 42 new orphan genes were identified per strain and plotting only these orphan genes generate a straight line confirming the addition of new genes by each sequential strain sequenced (Supplementary Figure S1B). These analyses are consistent with an active process of adaptation by the *Acinetobacter* strains with a constant acquisition of new genes by different strains and a more limited number of core sequences responsible for house-keeping functions. They also indicate that as more genomes are sequenced, more strain-specific genes are expected to be found, as well as other strains which share the so far strain-specific genes.

### MLST, Clonal Complexes and Phylogeny

With the availability of the full set of gene sequences available for the *Acinetobacter* strains investigated here, a MLST analysis was carried out for the 46 sequenced genomes in order to define how the various strains are related to each other. The Pasteur scheme Sequence Type (ST) identified a total of six *A. baumannii* STs circulating in the hospitals investigated and belonging to four clonal complexes previously reported as widespread among clinical isolates from this species: ST1 (5 strains, clonal complex CC1); ST15 (10 strains, CC15); ST25 (1 strain, CC113); ST79 (14 strains, CC79); ST113 (13 strains, CC113); and the new ST881 (CC1), which is represented by two strains having a *rpoB* single locus variant from ST1 (Supplementary Table S3). The *A. nosocomialis* strain (Acb_11) also displayed a previously non-described MLST profile with a new *fusA* allele and had a new ST assigned (ST882).

**FIGURE 1:**
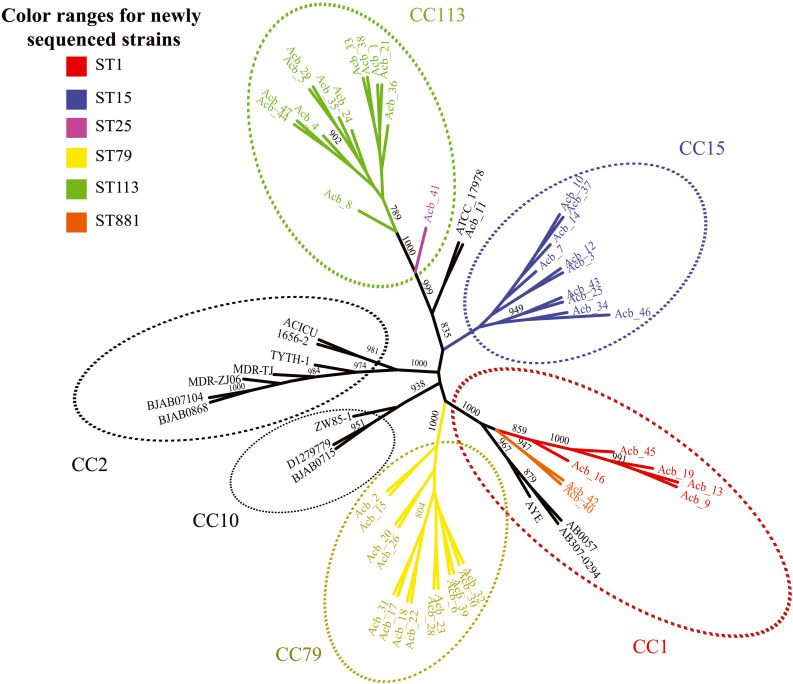
Phylogenetic evaluation of the genomes sequenced here and in comparisons with selected reference genomes. An unrooted tree was built with the core genome of all *Acinetobacter baumannii* strains sequenced in this study and selected reference published genomes. Colors scheme follow the sequence type (ST) determined in the Pasteur’s MLST scheme. The numbers near the branches are supporting bootstrap values with only the bootstrap values higher than 700 shown. Acb - *Acinetobacter baumannii*.

**FIGURE 2:**
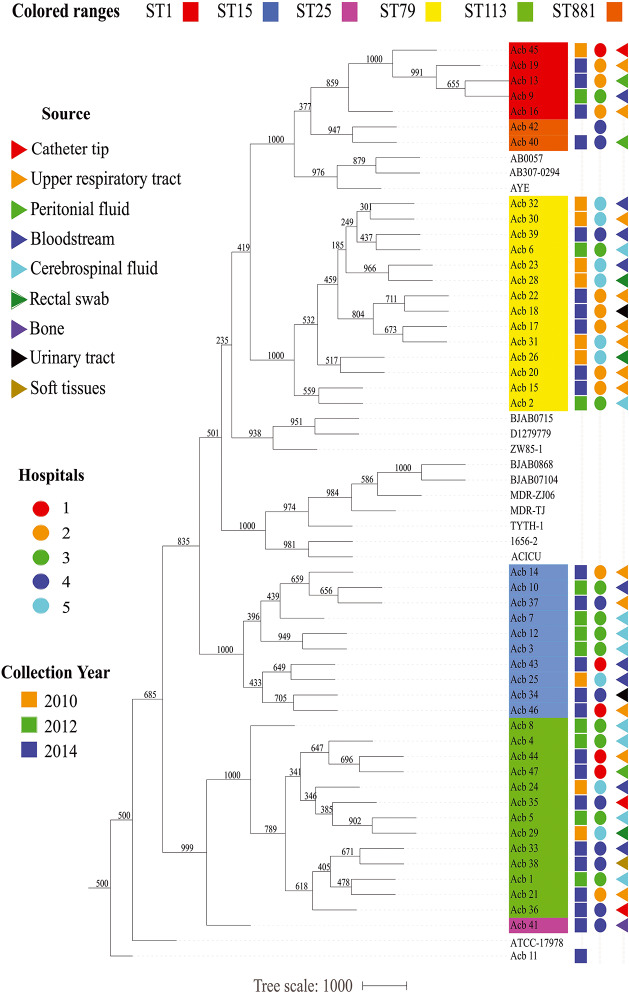
Epidemiological features of the *Acinetobacter* strains characterized in this study. The source of the bacterial samples, the hospitals from where the strains were isolated and the year of sample collections are displayed at the right and represented by colored triangles, circles or squares, respectively. A phylogenetic tree comparing all strains sequenced here, and with selected reference genomes, is included in the right, color coded as in Figure 1.

Next, we performed a phylogenomic analysis comparing the 46 genomes reported here with the first 14 *Acinetobacter* reference genomes. This analysis was based on an alignment-free phylogenetic tree created with the sequences of 1,740 core proteins common to all these 60 genomes. It revealed the existence of seven well supported monophyletic groups (bootstrap support > 800) corresponding to related clonal complexes (Figure 1), as previously defined (Karah et al., 2012). Two of these groups contained only references genomes belonging to CC2 and CC10 while a third group included seven of newly sequenced strains (five ST1-red and the two ST881-orange strains) and three reference genomes, all belonging to complex CC1. Comprising only strains sequenced here, two other groups were based on strains from CC79 (ST79-yellow in the figure) and CC15 (ST15-blue), while one more group, corresponding to CC113, included the thirteen ST113-green strains and the single ST25-pink strain (Acb_41). The seventh group included in the figure is represented by the *A. nosocomialis* Acb_11 plus the reference genome of *A*. *baumannii* ATCC 17978 (ST437), isolated from a French infant patient in 1951 (Smith et al., 2007). The phylogenetic analysis (also shown in Figure 2) highlights the CC1 strains as a basal group, with CC79 as its sister clade. Nearly all the *A. baumannii* reference strains included here were found within a larger clade, which also included the CC1 and CC79 strains from this study. The CC15 and CC113 *A. baumannii* strains were more divergent, with the CC113 strains forming a well-supported sister clade to all other confirmed *A. baumannii* strains.

The reduced number of single-copy orthologs or core genes conserved across all sequenced strains in this study, which in a few cases could be a possible consequence of the fragmented nature of the genomes, prompted us to investigate the degree of conservation/divergence in gene content seen between strains belonging to different STs, in order to identify possible lineage-specific genes. Indeed, a considerable, but variable, number of core genes were therefore found to be ST or lineage-specific (also shown in Supplementary Table S3). For instance, 164 genes were found common to all the ST1 strains that were not found in any of the *A. baumannii* strains belonging to other STs. Remarkably, for the ST15 (505 genes in common), ST79 (706 genes) and ST113 (897 genes), a much larger number of ST specific genes were seen, contrasting even with the *A. nosocomialis* strain, Acb_11, which has only 256 genes not found in the other strains investigated here. These numbers reflect the remarkable ability of the *A. baumannii* strains to acquire new genes which can be maintained in a ST (or clonal lineage) specific manner.

### ST Distribution, Date and Place of Isolation and Collection Method

Prior to any detailed genetic analysis, and based on the ST identification results, we next investigated any association between specific *A. baumannii* STs and variables such as date and place of isolation and collection methods. As summarized in the Figure 2, except for the strains from hospital 1, collected both in 2010 and 2014, bacterial samples from individual hospitals were generally collected from a single year so it is not possible to evaluate changes in frequencies for the various STs over different years for specific hospitals. Nevertheless, four out of six STs investigated here (ST1, ST79, ST113, and ST15) were collected over all the 3 years sampled (2010, 2012, and 2014), while the single ST25 and both ST881 *A. baumannii* strains were isolated only in 2014. *A. baumannii* strains belonging to ST113 and ST15 were isolated from all five hospitals studied, while ST79 grouped strains from four different hospitals and the ST1 strains are derived from three of the five hospitals. The same hospital where the single ST25 strain was isolated was also found to have strains from ST15, ST79, and ST113, as well as the two ST881 strains. The other four hospitals were found to harbor strains from three or four of the STs identified in this study. No correlation between the collection method used for strain isolation and the different STs was found. Overall, we can observe that despite the diversity in circulating *A. baumannii* STs identified in this study, all six STs have been simultaneously circulating at least since 2014, and multiple STs were found in each of the different hospitals investigated, with no clear indication of changes in ST frequency within the municipality during the time frame analyzed.

### Antibiotic Resistance Determinants and Related Genes

**FIGURE 3:**
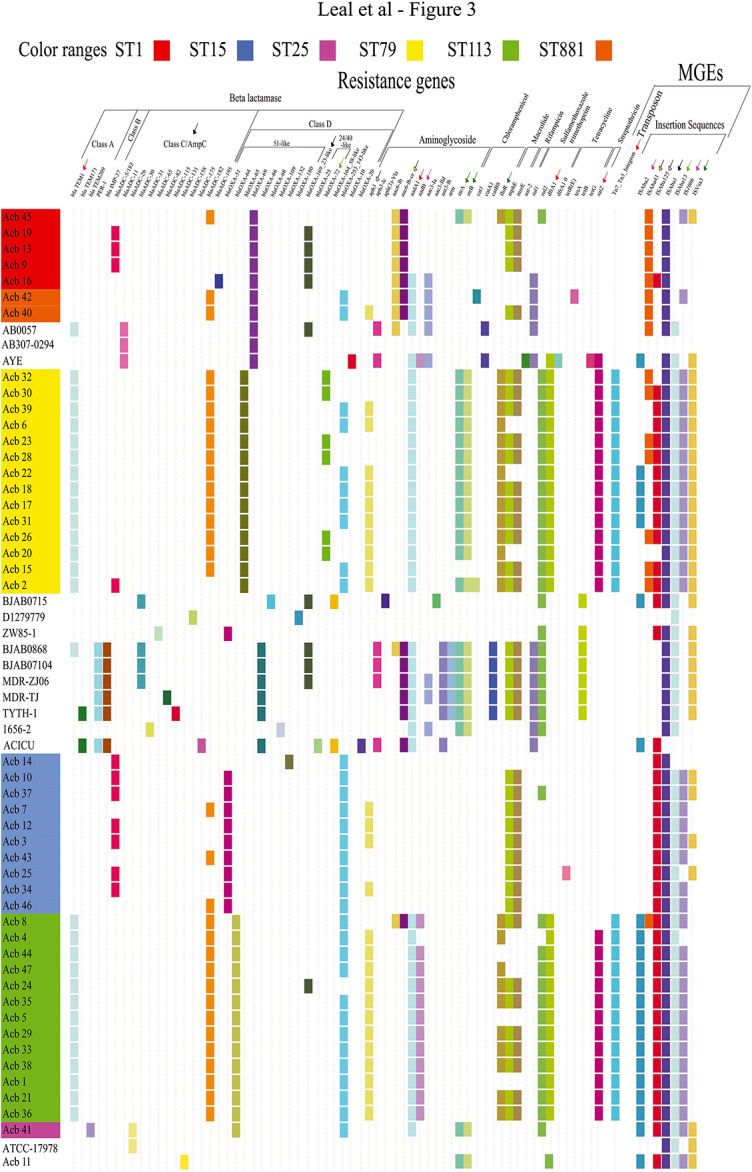
Overview of the resistance genes identified as well as selected mobile genetic elements (MGEs). The *Acinetobacter* strains are sequentially ordered according to their STs and the phylogenetic relationship previously defined (Figure 2 and Supplementary Figure S1) and color-coded as in Figure 2. The identification of the resistance genes was carried out with the sequences from the ResFinder database (*e*-value cutoff 1 × E^–5^, coverage ≥0.9, identity ≥0.9). A positive match in the figure is indicated by a colored rectangle, with different genes colored differently. Negative matches are shown as blank spaces. Resistance genes are sorted by antibiotic classes to which they confer resistance. A selection list of mobile genetic elements (MGEs) characterized by homology search with the ISFinder database and linked to specific resistance genes is also displayed in the right side of the figure (more detailed in Supplementary Figure S2), with the transposon separated from insertion sequences (IS) and these sorted according to the IS family to which they belong to. Similarly colored arrows above resistance genes and MGEs indicate the association found between them.

The diverse profile of antibiotic resistance observed for the strains studied here prompted us to perform a detailed analysis of the resistance associated genes present in the draft genomes evaluated in this study. These include intrinsic and acquired β-lactamases encoding genes and allelic variants, even though not all may be necessarily be required for resistance, as well as genes which confer resistance to aminoglycosides, fluoroquinolones, chloramphenicol, macrolides, rifampicin, trimethoprim/sulfamethoxazole and tetracycline (Figure 3). Strains assigned to ST79 and ST113 accumulated a higher number of antimicrobial resistance and related genes than ST1 or ST15. If only genes found in more than one strain of the same ST are taken into account, seven gene variants were found for ST1 and six for ST15 strains, compared to 15 and 13 genes seen for ST79 and ST113, respectively. Nevertheless, with the exception of the resistance phenotype to gentamicin, where the ST15 strains were markedly more susceptible to this drug than the strains from other STs, no significant increase in antimicrobial susceptibility was seen for the ST1 or ST15 strains when compared to the remaining STs studied here, as discussed below. Also, a further analysis of the distribution of the more abundant resistance genes from ST79 and ST113 strains, based on their mode of action, showed that there is no enrichment for any particular type of antimicrobial resistance. Instead, redundant genes seemed to occur multiple times in related strains. For instance, most ST79 strains had four β-lactamase and four aminoglycoside resistance genes, with, respectively, four and three genes also seen for most ST113 strains, while three β-lactamase and two or one aminoglycoside resistant genes were seen in multiple strains from ST1 or ST15. Importantly, several genes involved in resistance, or genes related to those, were commonly shared by both sets of ST79 and ST113 strains. This is relevant considering their greater divergence and the fact that these genes might be missing from ST1 and ST15 strains, more closely related to ST79. This observation suggests that selected genes were either being preferentially exchanged between strains belonging to ST79 and ST113 or being selectively acquired by these strains only.

For the β-lactamase encoding genes we opted to first investigate genes for the different Ambler classes independently of them having a defined role in resistance. For the Ambler class A β-lactamases, two genes were found in the strains sequenced in this study with a clear ST specific pattern for one of them, *bla*_TEM–1_. This gene was found within all genomes of the ST79 and ST113 strains but in none of the other STs, a first evidence indicating a preferential exchange of resistance genes between these two STs or independent acquisition events restricted to them. In contrast, the second class A β-lactamase gene, *bla*_TEM–209_, was found in the single ST25 strain (Acb_41). Two other class A genes, *bla*_TEM–171_ and *bla*_PER–1_, were found only in reference strains belonging to CC2. Regarding the class B β-lactamase genes, none were found in the strains from Recife, although one gene (*bla*_IMP–37_) was found in several reference strains belonging to the globally spread CC2 group and that also harbor the class A *bla*_PER–1_ gene. A single gene encoding an intrinsic class C *Acinetobacter*-derived cephalosporinases, the AmpC (or ADC) enzyme, was recovered from all *Acinetobacter* genomes analyzed here, including the reference strains and the one classified as *A. nosocomialis* (Acb_11), always associated with an ISAba1 insertion (discussed further in the last section). After comparisons with available databases (Karah et al., 2017; Naas et al., 2017), specific alleles were identified which were found to be differentially distributed among the various strains and were generally absent from the genomes used as references. The *bla*_ADC–182_ allele was by far the most widely distributed, found in 32 strains from five of the six *A. baumannii* STs identified (also shown in Figure 3 and detailed in Supplementary Table S4). This was the sole allele found for the ST113 and ST881 strains and it was also present in thirteen of the fourteen ST79 strains. The second most frequent allele, more closely related to *bla*_ADC–5_/*bla*_ADC–183_, was mainly found in the ST1 and ST15 strains and in Acb_11 (from ST79). Two other alleles were also found, *bla*_ADC–26_, in Acb_16 (ST1), and *bla*_ADC–195_, in the ST25 strain (Acb_41). Intrinsic class D β-lactamases genes *bla*_OXA–51_ or *bla*_OXA–51_-like were also detected in all *A. baumannii* strains sequenced, but for these a clear association was found between the allelic variant of the *bla*_OXA–51_-like identified and a specific ST or phylogenetic group, reinforcing the ST identification and phylogenetic analysis, although these genes are not likely to be associated with the resistance phenotype. For instance, *bla*_OXA–69_ was found in ST1 and in the related ST881 and reference strains, *bla*_OXA–65_ in ST79, *bla*_OXA–64_ in ST113 and *bla*_OXA–51_ in the ST15 strains.

With the exception of Acb_45, all strains, independent of ST, included one additional gene coding for a D class β-lactamase known to have a carbapenemase activity and which might be associated with a resistance phenotype, the acquired carbapenem-hydrolyzing class D β-lactamases (CHDL): most ST1 strains and one ST113 strain had the *bla*_OXA–169_; some ST79 strains only had *bla*_OXA–72_; and strains from five of the studied STs (ST15, ST25, ST79, ST113, and ST881) were found associated with *bla*_OXA–253_. The presence of the *bla*_OXA–253_ gene in strains from nearly all *A. baumannii* STs sequenced here but not in the reference genome sequences, indicates a recent spread of this more recently reported OXA-carbapenemase gene between the different STs or the spread of clones carrying this gene over different hospitals, as recently reported by some of us (de Sá Cavalcanti et al., 2017). Indeed, for ST79 only, the evidence indicates a chronological replacement of OXA-72 producing *A. baumannii* clinical isolates for OXA-253 producers in the more recent years, since most of the strains collected in 2010 have the acquired *bla*_OXA–72_, a variant of the *bla*_OXA–24_ gene, while those collected in 2012 and 2014 in general have *bla*_OXA–253_. Regarding cephalosporin resistance, when the resistance profile obtained for the various strains included in the present study was superimposed with the ST identification derived from the genome sequencing effort (summarized in the Supplementary Table S5), a mixed pattern was observed regarding antibiotic resistance and ST that depends on the drug evaluated. The only *A. baumannii* strain susceptible to all three cephalosporin drugs tested was the single ST25 strain investigated here, while the strains showing intermediate resistance to cefepime were mostly found among the ST15 and ST113 strains, contrasting with the ST1 strains that were all resistant to this drug. The sequenced data, however, reveals no clear explanation for these differences in the cephalosporin resistance profile.

A different picture emerged regarding the aminoglycoside resistance genes and their distribution among the strains from the different STs. Most of the ST1, ST881, and ST113 strains displayed resistance to the amikacin, with a mixed profile observed for both sets of ST15 and ST79 strains (Supplementary Table S5). In contrast, a clear association between ST and resistance was seen for the second aminoglycoside gentamicin, since all ST1, ST881, and nearly all ST113 (12/13) strains were resistant to this drug, while all ST15 strains were susceptible and only the ST79 strains were of a mixed phenotype. Genes encoding aminoglycoside modifying enzymes, including acetyltransferases, phosphotransferases, and adenylyltransferases were found distributed among the different phylogenetic groups and STs and these were more frequently found for strains belonging to ST79 and ST113. Indeed, an association between the presence of the *aph3* gene and resistance to amikacin was seen for nearly all ST15, ST79, and ST113 strains. The two exceptions were the ST79 Acb_28 and the ST113 Acb_08 strains, which lacked the *aph3* gene despite displaying resistance to amikacin. Acb_08, however, was the sole ST113 strain having both *aac6-Ib* and *aac6-Ib-cr* genes, also found in the ST1 and ST881 strains, all displaying some resistance to amikacin but missing *aph3*. In contrast, Acb_41, the single ST25 strain, also displayed some resistance to amikacin despite the absence of any of these three genes. For the second aminoglycoside, gentamicin, a clear correlation between the presence of the *aadB* gene, associated with resistance to this drug (Hamidian et al., 2012), was seen for the ST113 strains, but no similar correlation was observed for strains belonging to the other STs where the *aadB* gene was notably missing. For the aminoglycoside resistance, then, most but not all resistance phenotypes can be explained by the presence or absence of previously characterized genes in the sequenced genomes.

The QRDR (Quinolone-Resistance Determinant Region) of *Acinetobacter* strains was also analyzed in order to identify modifications in the genes for the DNA gyrase subunit A (*gyr*A) and topoisomerase IV subunit C (*par*C), which have been associated with high levels of fluoroquinolone resistance (summarized in the Supplementary Table S6). All 45 *A. baumannii* clinical strains had the GyrA modification S83L, associated with high level of ciprofloxacin resistance (Vila et al., 1994, 1997) and in agreement with their resistance profile to this drug. Other modifications encoding the G81C and E87G substitutions were observed in the *gyr*A gene from two strains displaying cross-resistance to levofloxacin, the ST1 Acb_16 and the ST113 Acb_21 strains, both isolated in 2014. The S80L mutation targeting the topoisomerase IV ParC was observed in most of the newly sequenced strains, with the exception of those belonging to ST79, but it does not correlate with any significant increase in resistance. For the ST79 strains, several of those had a S80Y ParC substitution that also does not correlate with levofloxacin resistance, while others had a E84K substitution that might be associated with a reduced susceptibility to this drug. However, since other ST79 strains lacking this substitution also displayed some resistance to levofloxacin, it does not fully explain the changes seen in resistance. Likewise, the resistant profile to levofloxacin observed for the Acb_40 (ST881), Acb_10 (ST15), and Acb_08 (ST113) strains is not associated with the mutations investigated in both *gyr*A and *par*C genes.

Several other antimicrobial resistance genes were identified in our search of the sequenced genomes but whose resistance profile to the corresponding antimicrobial agents are not available for the different strains, since we needed to focus on a selected set of tested drugs, chosen mainly for their clinical relevance. Nevertheless, genes associated with resistance to chloramphenicol, tetracycline and others were identified in the analysis carried out and the search results are shown in Figure 3. Many of those were differentially associated with the various STs and include several that were specifically present only in ST79 and ST113 (*sul2*, *dfrA1*, and *sat2*, for example), but were otherwise missing from nearly all other strains included in the analysis. Noteworthy, there were also two genes responsible for the resistance to the aminoglycoside streptomycin (*strA* and *strB*) which were selectively missing from the ST15, ST113, ST881, and nearly all ST1 strains.

### Selected Virulence Factors

**FIGURE 4:**
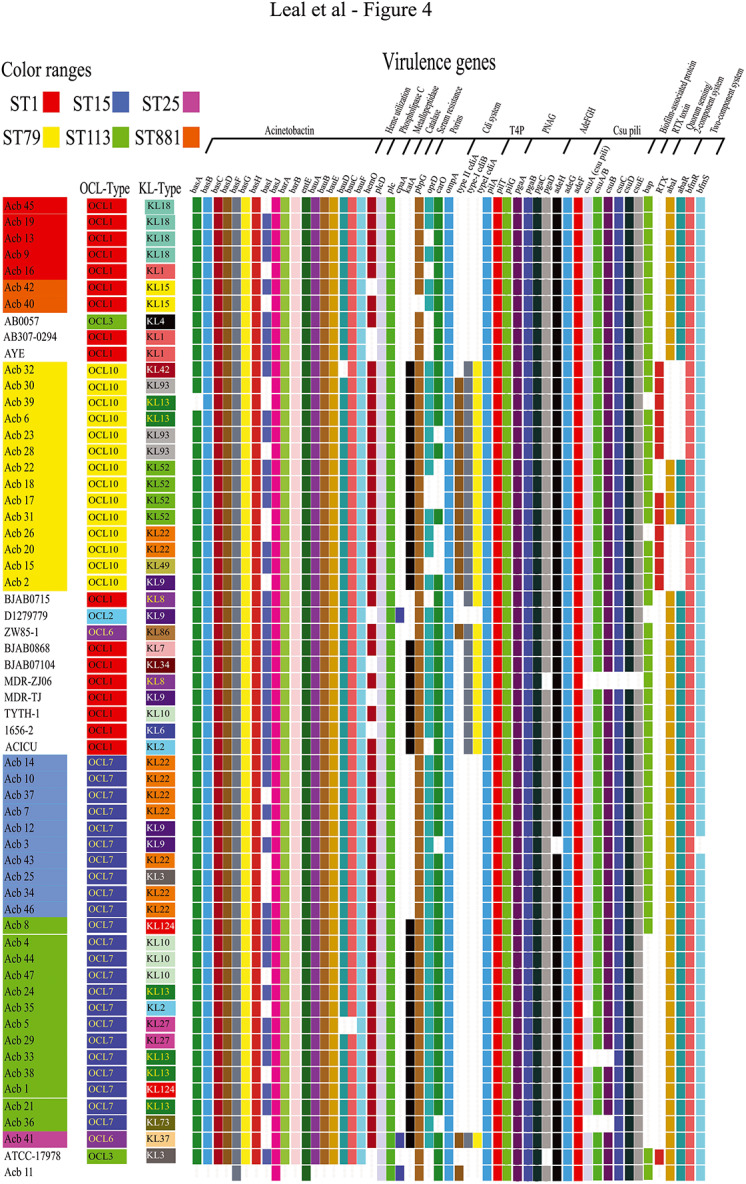
Overview of the virulence genes identified and definition of the OC/K loci for the strains investigated here. As for Figure 3, the *Acinetobacter* strains are also sequentially ordered according to their STs and the phylogenetic relationship previously defined (Figure 2 and Supplementary Figure S1), with a positive match indicated by a colored rectangle and negative matches are shown as blank spaces. The virulence genes were identified comparing the annotation made with PROKKA to Vfdb database (*e*-value cutoff 1E^–5^, coverage ≥0.9, identity ≥0.9). In the figure, these genes are sorted by the mechanistic feature that they likely confer to *A. baumannii* strains. The predicted *Acinetobacter* OCL and KL-types for each strain are also indicated in the figure and were defined as shown in the Supplementary Table S7.

Selected genes encoding factors from the *Acinetobacter* virulence factor database (VFDB) plus a few others were also investigated regarding their presence/absence among the newly sequenced strains, with the relevant results summarized in Figure 4. Acinetobactin genes cluster and *hemO*, related to iron uptake, were generally present in almost all strains investigated here, with the notable exception of the *A. nosocomialis* Acb_11 strain. Genes coding the phospholipases C and D were present in all strains, while the *cpaA* gene, encoding the secreted coagulation targeting metallo-endopeptidase (CpaA) and related to reduced coagulation of human plasma (Waack et al., 2018), was found only in *A. nosocomialis* strain (Acb_11) and the *A. baumannii* Acb_41. In contrast, the catalase gene (*katA*), which protects bacteria from superoxidants produced by leukocytes as a host defense mechanism (Sun et al., 2016), was present in most of the sequenced strains, with the exception of those from ST1/ST881 and ST15. Also found in all or nearly all strains investigated here was the *pbpG* (penicillin-binding protein) gene.

Porins such as CarO and OprD, channels for influx of carbapenems, and the outer membrane protein A (OmpA), were investigated here with other virulence factors. The *omp*A gene was found in all strains, but *carO* and *oprD* were alternatively absent from multiple strains belonging to ST1 (only *oprD*) and ST79 (both genes absent from different strains) as well as single strains from ST15 and ST113 (*carO* only), with the *oprD* also missing from Acb_11. Another virulence *locus* investigated was the one coding for the CDI system, but among the sequenced strains those from ST79 (and also the single ST25 strain, Acb_41) were unique in having these genes. Regarding type IV pili formation, also investigated here, all strains from this study as well as the reference strains had the three *pil* genes.

Multiple biofilm-related virulence genes known from *A. baumannii* were also investigated and, in general, genes encoding proteins involved in biofilm and pili formation, adherence, and quorum sensing were found in almost all the sequenced strains. Indeed, all strains from this study had the genes encoding the AdeFGH efflux pump, as well as the *pgaABCD* locus, required for intercellular adhesin synthesis, and the chaperone-usher assembly system of *csu* pili. In contrast, genes encoding the Biofilm Associated Protein (*bap*) were also investigated but could not be found in the single ST25 and in most ST113 strains. These genes, however, are complex in nature, with very long coding sequence containing variable repetitive regions (De Gregorio et al., 2015), and the fragmented nature of the genomes sequenced here would require a more detailed analysis to better define their diversity and distribution. Curiously, only strains from ST79 have confirmed genes encoding both Bap and the RTX-serralysin-like toxin, with the latter gene found in most, but not all ST79 strains, and in none of the other strains targeted in this study. Additionally, we searched for two quorum sensing genes (*abaI* and *abaR*), found to be missing only for some of the ST79 strains, and for the two-component system *bfmRS* (*rstBA*). These last two genes were found in nearly all sequenced strains, with the single exception of Acb_3, where the histidine kinase *bfmS* gene was absent.

### Lipopolysaccharide (LPS)/Lipooligosaccharide (LOS) Biosynthesis

The LPS/LOS component of the outer membrane of Gram-negative bacteria is a major virulence component whose synthesis is dependent on several biosynthetic pathways (Wang and Quinn, 2010). As expected, the locus responsible for the synthesis of its hydrophobic lipid component, the conserved lipid A (*lpxABCDLM*), was present in all sequenced strains, with the single exception being Acb_40 where the *lpxA* gene was not found (not shown). No WaaL ligase was found in any of the sequenced strains but, with the exception of Acb_11 and Acb_31, all other sequenced strains had the PglL *O*-oligosaccharyltransferase enzyme, a putative substitute for WaaL ligase (Kenyon and Hall, 2013). Curiously, only in ST113 and ST25 the *pglL* gene open reading frame is complete having its three domains: pglLA, wzyC, and wzyC2. In strains from other STs, either pglLA plus wzyC or wzyC alone are found.

The variable carbohydrate component of LPS/LOS is encoded by the outer core *locus* (OC), which comprises genes involved in the synthesis, assembly and export of complex oligosaccharides that are then linked to lipid A to form the LPS/LOS (Wang and Quinn, 2010; Kenyon and Hall, 2013). The OC *locus* (OCL) is flanked by the *ilvE* (aminotransferase) and *aspS* (aspartate-tRNA ligase) genes and both are found in all sequenced genomes. Different OCL variants have been reported, which vary according to the presence or absence of genes encoding multiple glycosyltransferases and other enzymes (Kenyon et al., 2014). The complete OC gene cluster was not contiguously assembled for all strains sequenced here, although their OCL-type could be tentatively defined using recently described tools (Wick et al., 2018; Wyres et al., 2020), as summarized in Figure 4 and also in the Supplementary Table S7. All the analyzed *Acinetobacter* CC1 strains (from ST1 and ST881) had the OCL1 variant, while all the ST79 strains were found to have the OCL10 type from Group A. In contrast, OCL variants belonging to Group B were found for the remaining sequenced strains with the ST15 and ST113 strains, and also the *A. nosocomialis* Acb_11, having OCL7, while the single ST25 strain had the OCL6 variant. Overall, the OCL types observed for the sequenced strains were tightly linked to their ST-classification as well as their phylogenetic relationships, with different types generally associated to strains from different STs, the exception being those belonging to the ST15 and ST113 strains.

### Genes Associated With the K Antigen Synthesis

The K *locus* also determines the production of complex oligosaccharides units that are exported to the outer membrane of Gram-negative bacteria and includes genes responsible for the synthesis of the exopolysaccharide capsule, the K antigen (Iwashkiw et al., 2012; Kenyon and Hall, 2013; Senchenkova et al., 2015; Holt et al., 2016; Shashkov et al., 2016, 2017; Kenyon et al., 2017, 2019). Despite having the flanking *fkpA* and *lldP* genes, none of the strains sequenced here exhibited a unique and fully assembled K locus contig (not shown). Module A (*wza*, *wzb*, and *wzc*) is nevertheless present in all strains sequenced here, as well as the *gna* gene, positioned immediately after it, and module B (*galU*, *udg*, *gpi*, *gne1*, *pgm*, and *lldP*). The KL gene cluster was also evaluated and found within multiple contigs of the draft genomes, with several genes appearing to be interrupted by frameshifts and/or insertion sequence. It was not always possible to clearly define distinct KL groups for the sequenced strains and these would require complementary studies. However, a tentative classification was also made based on the presence/absence of previously defined genes, as described for the OCL-types (summarized in Figure 4 and in the Supplementary Table S7).

Four of the five ST1 strains likely have the KL18 cluster, with the fifth strain, Acb_16, having genes belonging to KL1, a closely related cluster, and both ST881 strains having KL15. The ST79 strains are much more diverse, with seven different KL-types identified for the 14 strains: KL9 (Acb_2), KL13 (Acb_6 and Acb_39), KL22 (Acb_20 and Acb_26), KL42 (Acb_32), KL49 (Acb_15), KL52 (Acb_17, Acb_18, Acb_22, and Acb_31), and KL93 (Acb_23, Acb_28, and Acb_30). For the ST15 strains, three KL types were identified, with seven strains found having the KL22 (Acb_7, Acb_10, Acb_14, Acb_34, Acb_37, Acb_43, and Acb_46), two with KL9 (Acb_3 and Acb_12), and one with KL3 (Acb_25). Six KL-types were found for the ST113 strains: KL2 (Acb_35), KL10 (Acb_4, Acb_44, and Acb_47), KL13 (Acb_21, Acb_24, Acb_33, and Acb_38), KL27 (Acb_5 and Acb_29), KL73 (Acb_36), and KL124 (Acb_1 and Acb_8). Lastly, the single ST25 strain, Acb_41, was classified as having the KL37-type cluster. The ST79 and ST113 strains then are more variable in terms of KL-types when compared with the other STs. The data also highlights the KL-types shared by newly sequenced strains belonging to more than one of ST, such as KL22, found in both ST79 and ST15 strains, and KL13, found in strains belonging to the more distantly related ST79 and ST113 strains.

### Mobilome

Searches for known mobile genetic elements (MGEs), including genomic islands, insertions sequences and transposons, were also carried out here using the available sequences (results summarized in Figure 3 and Supplementary Figure S2). Considering the genomic islands, we first investigated regions mapping to AbaR0-type islands [recently reviewed by Hamidian and Hall (2018)]. In addition to two of the reference CC1 strains (AB0057 and AYE), segments belonging to these islands were clearly found in all ST1 and in both ST881 strains sequenced here (Supplementary Figure S3A), with disrupted *comM* gene sequences. When only the AbaR0 backbone is considered, based on the Tn*6019*::*Tn6018* transposons, most assemblies were fragmented into one or more contigs, missing several segments, with the exception of those from Acb_19 and Acb_13 (detailed in the Supplementary Figure S3B). Three ST79 strains (Acb_22, Acb_18, and Acb_17) also have a disrupted *comM* gene split by an AbaR0-like prototype, with only six genes related to transposases as well as two stress related genes (also shown in Supplementary Figure S3B). Regions homologous to AbGRI1-type islands (Nigro et al., 2013; Zhu et al., 2013; Blackwell et al., 2016) displayed a more dispersed distribution among the sequenced strains, with contigs bearing several genes originally mapped to this island found in all five ST1 strains as well as in several ST15 (8/10), ST79 (3/14), and ST113 strains (3/13) (Supplementary Figure S2 – genomic islands). A major difference between the ST1 and ST881 strains then was the absence of the AbGRI1-type segments from the ST881 strains. More detailed information about the genes found associated to these islands is included in Supplementary Figures S4, S5. Regarding the other sequenced genomes, most of them harbor an intact *comM* gene and no regions homologous to the investigated islands.

Next, a search for composite transposons revealed a single Tn*3* and two Tn*7* transposons plus a chimeric transposon having both Tn*3* and Tn*7* features. These were found distributed among the different genome sequenced with a substantial variation in copy number, from 27 to 81. The Tn*3* element (MGEs section from Figure 3 and Supplementary Figure S2) was found in some of the reference genomes as well as in all the ST79 and ST113 strains and in one of the two ST881 strains (Acb_42). In contrast, the first Tn*7* transposon was found in all the genomes analyzed here, including the *A. nosocomialis* strain, with a higher copy number for ST79. The second Tn*7* transposon was also found in all ST1, ST15, ST25, and ST881 strains and in most of the reference genomes but was missing from several of the ST79 and ST113 strains. The fourth transposon element, having both Tn*3* and Tn*7* features, however, showed a more restricted distribution and was found strictly in the ST79 and ST113 strains (see the following section).

A total of 18 insertion sequences (IS) from 10 different families were also found in the different genomes (Supplementary Figure S2): IS*1*, IS*3*, ISL*3*, IS*4*, IS*5*, IS*6*, IS*30*, IS*66*, IS*91*, and IS*256*. Those ISs varied in copy number from 6 to 14. They also varied in distribution with some being widely distributed among the genomes investigated, such as ISAba1, and others much more restricted, including ISAba17, which is restricted to ST113 strains, and ISAba31, mostly found in ST15 strains.

Regarding phage related sequences, complete or almost complete prophage genomes were detected in several of our samples. Examples are the genomes for the Haemop_SuMu_NC_019455 phage, found in 38 of the 47 strains, and those for the Acinet_Bphi_B1251_NC_019541 phage, found in 41 of the 47 genomes sequenced here (data not shown). Since virulence or resistance genes were not found to be associated with any of the phages detected, we will not discuss them further in this manuscript.

### Genetic Basis for the Transfer of Resistance Determinants

**FIGURE 5:**
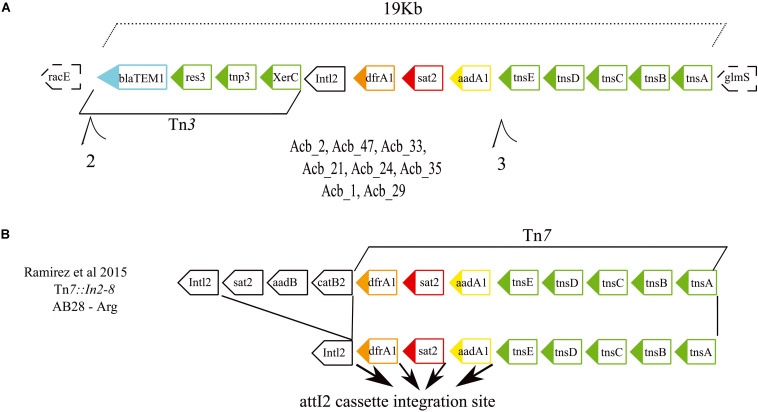
Gene structure of the novel Tn*7*-Tn*3* transposable element. **(A)** Schematic representation of the integron discovered in this study formed by the fused Tn*7* and Tn*3* transposons found in the American STs 79 and 113. The dashed boxes correspond to the integron flanking genes. **(B)** Schematic representations of the previously described Tn*7* transposons (Ramirez et al., 2005) related to the one reported here. Such integrons carry four resistance genes highlighted in light blue, orange, red, and yellow. Genes responsible for the transposition/recombination process are in green. Black arrows denote the *attl2* cassette integration site.

A search was also carried out to investigate specific associations between the various resistance genes and different MGEs, aiming to clarify how the antibiotic resistance genes were being affected by these elements (results summarized in Figure 3 and also in Supplementary Figure S2). The first MGE linked to resistance genes here was the novel Tn*7*-Tn*3* element found to be specifically associated with the ST79 and ST113 strains. A more detailed analysis of its sequence revealed that its 3′ region includes a 12 Kb Tn*7* transposon harboring three hypothetical ORFs and five genes related to the Tn*7* transposition process plus a 5′ integron consisting of three resistance gene cassettes (*drfA1*, *sat2*, and *aadA1*) and the *Intl2* gene. These are essentially identical to an MGE previously reported from Argentina (Ramirez et al., 2005), but the element identified here is missing the 5′ end of the previously described element, consisting of three ORFs encoding resistance genes. In its place a novel 5′ region ∼6.9 kb in length is found, which harbors three genes related to a Tn*3* transposition machinery, a *bla*_*TEM1*_ resistance gene and two hypothetical ORFs (Figures 5A,B). At least eight strains (one from ST79 and seven from ST113) have this element inserted in a known hotspot for Tn*7* transposons, between genes *racE* (Glutamate racemase 1) and *glmS* (Glutamine–fructose-6-phosphate aminotransferase). Contigs encompassing the full length ∼19 Kb element, but missing *racE* and *glmS* flanking genes, were also generated from thirteen other strains from ST79 and ST113. Only fragments of this integron, split into many contigs, were found for the remaining strains from these two STs, probably due to the lack of proper assembly, but again the presence of the integron sequences remained restricted to both these STs only.

**FIGURE 6:**
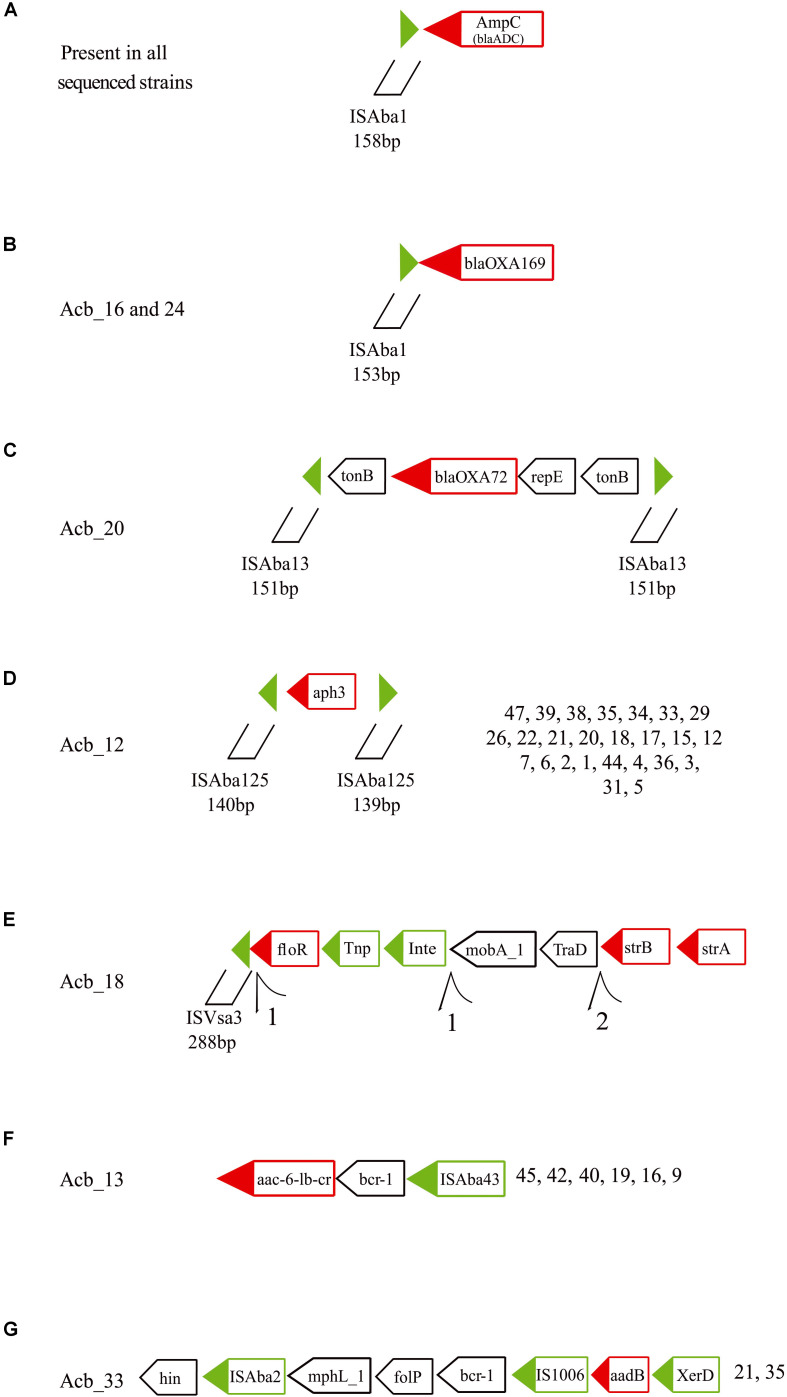
Schematic representation of other resistance genes associated with transposable elements found in the *A. baumannii* strains sequenced in this study. Green triangles are inverted terminal repeats of insertion sequences flanking resistance genes. Green boxes are genes related with the transposition process and red boxes are resistance and virulence genes. *Tnp*, transposase; *Int*, Integrase; and *XerD*, tyrosine recombinase. Further structures of selected resistance genes and associated TEs can be found in the Supplementary Figure S6. **(A)** The *ampC* (*bla*_*ADC*_) gene and its upstream ISAba1. **(B)** The *bla*_**OXA–169**_ gene with the identified upstream ISAba1. **(C)** The *bla*_**OXA–72**_ gene with flanking genes and ISAba13 insertions. **(D)** The *aph3* gene with flanking ISAba125 fragments. **(E)** The *strB*, *strA* and *floR* genes with associated ISVsa3 motifs. **(F)** The *acc-6-lb-c* and *bcr-1* genes and their association with ISAba43. **(G)** The *aadB* and proximal genes in association with ISAba2 and IS1006.

At least seven others different MGEs have some association to resistance genes. All 46 strains sequenced have an ISAba1 insertion upstream of the *ampC* (*bla*_*ADC*_) gene (Figure 6A, indicated by black arrows in Figure 3), a carbapenem resistance gene. Moreover, an ISAba1 insertion could also be identified upstream of another carbapenem resistant gene variant the *bla*_*OXA–169*_ gene (Figure 6B, also indicated in Figure 3) found in Acb_16 (ST1) and Acb_24 (ST113). Yet another carbapenem resistance gene linked to a MGE is the *bla*_*OXA–72*_ (a *bla*_*OXA–24*_ gene variant), encoding a OXA-72 carbapenemase, associated with a replication initiation gene (*repE*) and flanked by two transporter (*tonB*) genes and two ISAba13 insertion sequences 151 bp in length (Figure 6C, light green arrows in Figure 3). Those IS could not be identified as full length elements (IS containing coding region and terminal repeats) and hence were fragmented at the end of assembled contigs, since the limited size of the sequenced fragments and the presence of multiple identical elements prevented an adequate assemblage of the fragments downstream. However, their 151 bp extremities were intact, indicating that this composite IS is probably capable of mobilization in the presence of a transposase *in trans* encoded by any coding full-length element inserted in the genome and independently of any transposases being encoded by a downstream ORF.

Twenty-six strains from three different STs (ST15, ST79, and ST113) showed the aminoglycoside resistance gene *aph3* flanked by two ISAba125 fragments ∼140 bp long, also suggesting a potential mobilizable unity in the presence of transposases (Figure 6D, light gray arrows in Figure 3). In Acb_18 (ST79), a cassette of three resistance genes (*strB*, *strA*, and *floR*), the first two of which are associated with resistance to streptomycin, were found in association to an integrase, a transposase and a 5′ ISVsa3 motif 288 bp long (Figure 6E, dark green arrows in Figure 3). Several rearrangements and fragmented contigs related to this cassette were also observed, mainly in assemblies from other ST79 strains (exemplified in the Supplementary Figure S6A). Another aminoglycoside resistance gene, *acc-6-lb-cr*, was found associated with an ISAba43 in all ST1 and ST881 strains (Figure 6F, yellow arrows in Figure 3) and the same is also likely the case for a single ST113 strain (Acb_8), but here the sequence data is incomplete. In between the *acc-6-lb-c* gene and ISAba43 one more antibiotic resistance gene was always found, *bcr-1*, and indeed seven other strains belonging to ST79 have the *brc-1* gene next to the ISAba43 insertion in the absence of *acc-6-lb-c* (Supplementary Figure S6B). In addition, the 5′ upstream region of the *brc-1* gene is flanked by a mutator transposase coding sequence, reinforcing that there is a distinct *brc-1*/ISAba43 specific structure within the genomes from these strains. Yet another aminoglycoside resistance gene, *aadB* found only in ST113 strains, was identified with two insertion sequences, *ISAba2* and *IS1006* (Figure 6G, pink arrows in Figure 3), along with the gene encoding the tyrosine recombinases XerD and also a *brc-1* gene (Supplementary Figure S6C).

The contigs assembled here for the various *A. baumannii* strains, using short reads, were generally not long enough to produce or identify complete or near-complete plasmids associated with the sequenced strains. For the *bla*_OXA–253_ gene, however, whose genetic environment has been studied in more detail in these strains and has been the focus of a separate publication by us, a plasmid localization for this gene from most, if not all, strains is validated by the analysis carried out, since contigs encompassing up to 97% of the previously described plasmid were found (de Sá Cavalcanti et al., 2017). This is compatible with the broad distribution of the *bla*_OXA–253_ gene among five of the six STs investigated here, with the sole exception of ST1. Nevertheless, at this stage, plasmid localization could not be implied from the sequenced genomes for other genes known to be plasmid-based.

## Discussion

The data reported here confirms that at least four different CCs are circulating in the different hospitals from a single metropolitan area from Northeastern Brazil, with no obvious association found between any of the CCs/STs and variables such as hospitals, collection years or sampling methods. This is in contrast with other studies which usually reports outbreaks of either a single ST or closely related ones (Mosqueda et al., 2013; Wright et al., 2014; Li et al., 2015; Ou et al., 2015), although a recent report from Latin America has also found multiple lineages of MDR *A. baumannii* coexisting within the same hospital (Graña-Miraglia et al., 2020). Among those CCs founds here, CC1, CC15, and CC113 (ST25) are considered globally spread *Acinetobacter* clonal complexes, having been isolated from different parts of the world for more than 20 years (Diancourt et al., 2010; Di Nocera et al., 2011; Di Popolo et al., 2011; Karah et al., 2012; Sahl et al., 2015; Gaiarsa et al., 2019). In contrast, CC79 strains have been described mostly from North and South America but also in Europe (Karah et al., 2012; Villalón et al., 2013; Chagas et al., 2014; Wright et al., 2014; Bado et al., 2018; Da Silva et al., 2018; Levy-Blitchtein et al., 2018; Graña-Miraglia et al., 2020), while ST113 (also belonging to CC113) was described originally in the Middle East but has more recently mostly been reported from South America (Bonnin et al., 2013; Clímaco et al., 2013; Girlich et al., 2014). Here, the availability of draft genome sequences from multiple strains from various STs and clonal complexes co-existing in the same environments allowed a large-scale comparison of the multiple elements associated with their antibiotic resistance and virulence. Our results confirm previous analyses highlighting the diversity in *A. baumannii* gene content and in antibiotic-resistant genes as well as the high efficiency with which this bacteria acquires novel genetic elements (Imperi et al., 2011; Liu et al., 2014; Wright et al., 2014; Chan et al., 2015). It also suggests that the ability to exchange certain genetic elements between strains is not uniform for all STs, with strains from some STs being more prone to exchange these elements than others, as discussed below.

Multidrug resistant *A. baumannii* is a serious threat to immunologically compromised and critically ill patients under intensive care worldwide (Nowak and Paluchowska, 2016; Wong et al., 2017). This is also the case in Brazil and researchers have been using PCR based detection methods in order to access the resistance gene profile of hospital isolates (Adams-Haduch et al., 2008; Clímaco et al., 2013; Chagas et al., 2014; Da Silva et al., 2018) and even in isolates from environmental samples (Turano et al., 2016). Accumulation of resistance determinants to multiple classes of antimicrobials was observed among the genomes sequenced here, some clearly capable of being transferred between strains, independent of their ST, while others more associated with specific STs. Indeed, four resistance genes (*frA1*, *sat2*, *aadA1*, and *bla*_TEM–1_) were found to be inherited together due to the new MGE found here only in ST79 and ST113 strains and related to an element described from Argentina, carrying six resistance genes (Ramirez et al., 2005). Likewise, a wide distribution was observed for the *bla*_OXA–253_ gene, more recently described from Honduras, Brazil, and Peru (Girlich et al., 2014; Zander et al., 2014; Levy-Blitchtein et al., 2018). This gene, most likely plasmid encoded, was found among different STs comprising >70% of the *A. baumannii* strains sequenced here, as previously reported (de Sá Cavalcanti et al., 2017). Indeed, the recent expansion of the *bla*_OXA–253_ gene and its replacement of *bla*_OXA–72_ could be more clearly observed here in the ST79 strains isolated from different time periods, highlighting the speed with which these MGEs and associated resistance genes can be acquired. In contrast, the data available suggests that the genomic islands, although present in many of the strains sequenced here, might not be the main determinant for the spread of resistance, due to the lack of identifiable resistance genes in the islands found here. This is even more so for many of the ST79- and ST113-resistant strains, where genomic islands could not be found, in agreement with what has been previously reported with ST79 strains from North America (Wright et al., 2014).

In most instances the genetic analysis carried out here is consistent with the antibiotic resistance profile observed for the strains included in the present study. The presence of different *bla*_OXA–51_-like allelic variant associated with specific STs is in agreement with previous reports, where, *bla*_OXA–69_ has been found in ST1 strains, for example, while *bla*_OXA–69_ is associated with ST79, *bla*_OXA–51_ with ST15 and *bla*_OXA–64_ with ST25 (Karah et al., 2012). As expected, an acquired class D β-lactamase gene was nearly always found in all carbapenem resistant strains, such as *bla*_OXA–72_, *bla*_OXA–253_ and so on. Other genes associated with the aminoglycoside and fluoroquinolone resistance profiles were also observed for nearly all strains sequenced. However, some cases of antibiotic resistance in strains closely related to susceptible ones could not be linked to specific genes and these should be better investigated, since they might be associated with novel, yet uncharacterized, resistance mechanisms.

A noteworthy observation reported here is the identification of specific MGEs, as well as virulence and resistance genes, shared between the ST79 and ST113 strains only. Strains from ST113 are more distantly related from the ST79 strains than, for instance, the ST1 strains. However, the new Tn*7*-Tn*3* element, with associated resistance genes, is found only in the ST79 and ST113 strains. Likewise, the related KL13 genes associated with the K antigen synthesis, also likely interchanged between different *A. baumannii* strains, are only found in strains from these two STs and in none of the others investigated here. Other MGEs, however, are more widely distributed among the sequenced STs and more promiscuous, such as the MGE associated to *bla*_OXA–253_ (de Sá Cavalcanti et al., 2017). Even then, however, there is a noticeable absence of this gene from the ST1 strains, some isolated in the same hospital and year than some of the ST79 ones. These results suggest selective gene exchanges between strains from specific STs, perhaps associated with the presence of specific shared genetic elements. Indeed, a search for genes common only to strains from ST79 and ST113 revealed 159 genes shared by strains from these two STs which are not found in the other genomes investigated here. In contrast, only 34 genes are found in common between the ST79 and the more closely related ST1, with 29 genes in common between ST113 and ST1. It is possible then that among the genes shared only by the ST79 and ST113 strains there might be some involved with specific genetic exchange mechanisms that need to be further investigated.

Overall, our data revealed that in Recife, hospital physicians might be dealing with *A. baumannii* outbreaks of multiple clonal complexes, and which vary substantially in their resistance and virulence profile. The trend observed for the recent spread of the *bla*_OXA–253_ gene and maybe the genes associated with quinolone resistance, associated with the continuous increase in bacterial resistance in the clinics, further restricts the therapeutic options available for empirical treatment and the chance of achieving clinical success in infections caused by *A. baumannii*. This study emphasizes once again the need for the diversity in *A. baumannii* resistance mechanisms to be carefully considered in future monitoring strategies and in the decision-making regarding patients’ treatment by practicing physicians. It also highlights the need for further monitoring of *A. baumannii* isolated from the hospitals through the use of genetic methods, in addition to phenotypic tests.

## Data Availability Statement

The datasets generated for this study can be found in the https://www.ebi.ac.uk/ena/browser/view/PRJEB12754.

## Author Contributions

NL, AA, MM, TL-B, and Od-M-N contributed to the conception and design of the study. DX and IR carried out the antimicrobial susceptibility testing. CM-M and FS did all DNA extraction and the *bla*_OXA–51_ gene amplification while CD, CM-M, and FS performed the whole-genome sequencing. AR and TC organized and curated the database and performed the initial bioinformatics analysis. CM-M, CC, CD, DX, DV, FS, GW, IR, LA, NL, MB, and MA-F contributed with the identification and analysis of different genes or gene sets. AR, CD, DX, FS, TC, and GW wrote sections of the preliminary document. Od-M-N reviewed and edited the manuscript. All authors reviewed and approved the submitted version.

## Conflict of Interest

The authors declare that the research was conducted in the absence of any commercial or financial relationships that could be construed as a potential conflict of interest.

## Footnotes

1 https://www.ebi.ac.uk/ena/browser/view/PRJEB12754

2 http://www.bioinformatics.babraham.ac.uk/projects/fastqc

3 http://bioinformatics.net.au/software.velvetoptimiser.shtml

4 http://pubmlst.org/abaumannii/

5 http://www.paidb.re.kr/view_pai_from_genome.php?pa=NC_021729_R1

## Supplementary Material

FIGURE S1 Evaluation of overall gene content from the genomes sequenced in this study. **(A)** Cumulative curve of *Acinetobacter baumannii* pan-genome using as proxy the number of new ortholog group of genes detected by ORTHOMCL software with 100 replicates. **(B)** Cumulative graph showing the number of unique genes after the addition of each new strain, 1,000 replicates.

FIGURE S2 Full list of mobile genetic elements (MGEs) characterized by the homology search with ISFinder database are displayed in the right side of the figure and including genomic islands, transposons and insertion sequences (IS), the latter sorted according to the IS family to which they belong to. Since the MGEs can be found in multiple copies in each genome, the width of the colored rectangles reflects the number of copies found for each. The STs follow the colors displayed in Figure 1.

FIGURE S3 Regions from AbaR0 genomic islands found in the five ST1 and two ST881 strains. **(A)** Circular plot with regions with high identity from pair wise Blast comparisons between a reference AbaR0 island first detected in a *A. baumannii* strain from Australia (Hamidian et al., 2014) and the contigs derived from the sequenced Recife strains. **(B)** Striking examples of the AbaR0-like island dynamics found in a single contig flanked by the *comM* gene. A schematic representation of the AbaR0 island backbone transposons found in Acb_19 and Acb_13 (both ST1 strains) as well as Acb_22, Acb_18, and Acb_17 (all from ST79) is shown. Green boxes are MGEs related genes, red boxes define the resistance related genes, and yellow boxes represent the genes found in the islands sequenced in this study, but which are absent from the original AbaR0. It is important to note that, unless stated, this representation of the segments homologous to the AbaR0 and AbGRI1 islands found in the sequenced strains should not be interpreted as contiguous contigs. Only the presence or absence of genes originally found in an island should be considered. Any new gene present only in the newly sequenced strains will not be depicted in this figure.

FIGURE S4 Regions from AbGRI1 genomic islands found in ST1 and ST79 strains. Only the presence or absence of genes originally found in an island should be considered. Any new gene present only in the newly sequenced strains will not be depicted in this figure.

FIGURE S5 Regions from AbGRI1 genomic islands homologs found in ST113 and ST15 strains. Only the presence or absence of genes originally found in an island should be considered. Any new gene present only in the newly sequenced strains will not be depicted in this figure. Based on this analysis, for the ST113 strains, only Acb_21, Acb_33, and Acb_38 strains were considered to have true segments of an AbGRI1 island.

FIGURE S6 Other resistance and virulence genes found associated (in the same contigs) with transposition related genes and their assembly variability among the strains sequenced in this study. **(A)** Variations associated with the streptomycin (*strB* and *strA*) resistance genes, linked or not to ISVsa3 and transposable elements. **(B)** Variations associated with the *bcr-1* gene and the ISAba43 insertion. **(C)** Alternative profiles for the elements flanking the *aadB* resistance gene.

TABLE S1 Summary of the antimicrobial susceptibility profile exhibited by the single *Acinetobacter nosocomialis* and 45 *Acinetobacter baumannii* clinical strains investigated here against 11 different antimicrobial agents using the CLSI broth microdilution method. The background colors are derived from Figure 2 and represent the different STs to which the 45 *A. baumannii* clinical strains belong.

TABLE S2 Summary of the assembly and annotation statistics for the genomes from all strains sequenced in this study.

TABLE S3 Summary of the ST identification data for all strains sequenced in this study, with counts for shared single copy orthologs or lineage-specific core genes.

TABLE S4 Summary of the *bla*_*ADC*_/*ampC* identification analysis data for all strains sequenced in this study, comparing the identified alleles with the *bla*_OXA–51_-like alleles.

TABLE S5 Comparison of the ST identification and antimicrobial susceptibility profile for all 46 *A. baumannii* strains sequenced here.

TABLE S6 Mutations associated with the quinolone-resistance determinant region for the sequenced *Acinetobacter* strains.

TABLE S7 Summary of the gene identification data for the OC and K antigen gene clusters for the various sequenced *Acinetobacter* strains with definition of likely OCL and KL-types. The relevant output results from the *Kaptive* tool (Wick et al., 2018) using the curated databases of annotated reference sequences for *A. baumannii* K and OC loci (Wyres et al., 2020) were added to the table with the relevant OCL and KL-types listed, as shown also in Figure 4.
