# Supplementary figures and images for: Comparative Genomics of Acinetobacter baumannii Clinical Strains From Brazil Reveals Polyclonal Dissemination and Selective Exchange of Mobile Genetic Elements Associated With Resistance Genes
Source: Front Microbiol. 2020 Jun 17;11:1176. doi: 10.3389/fmicb.2020.01176 (PMC7326025; doi:10.3389/fmicb.2020.01176)

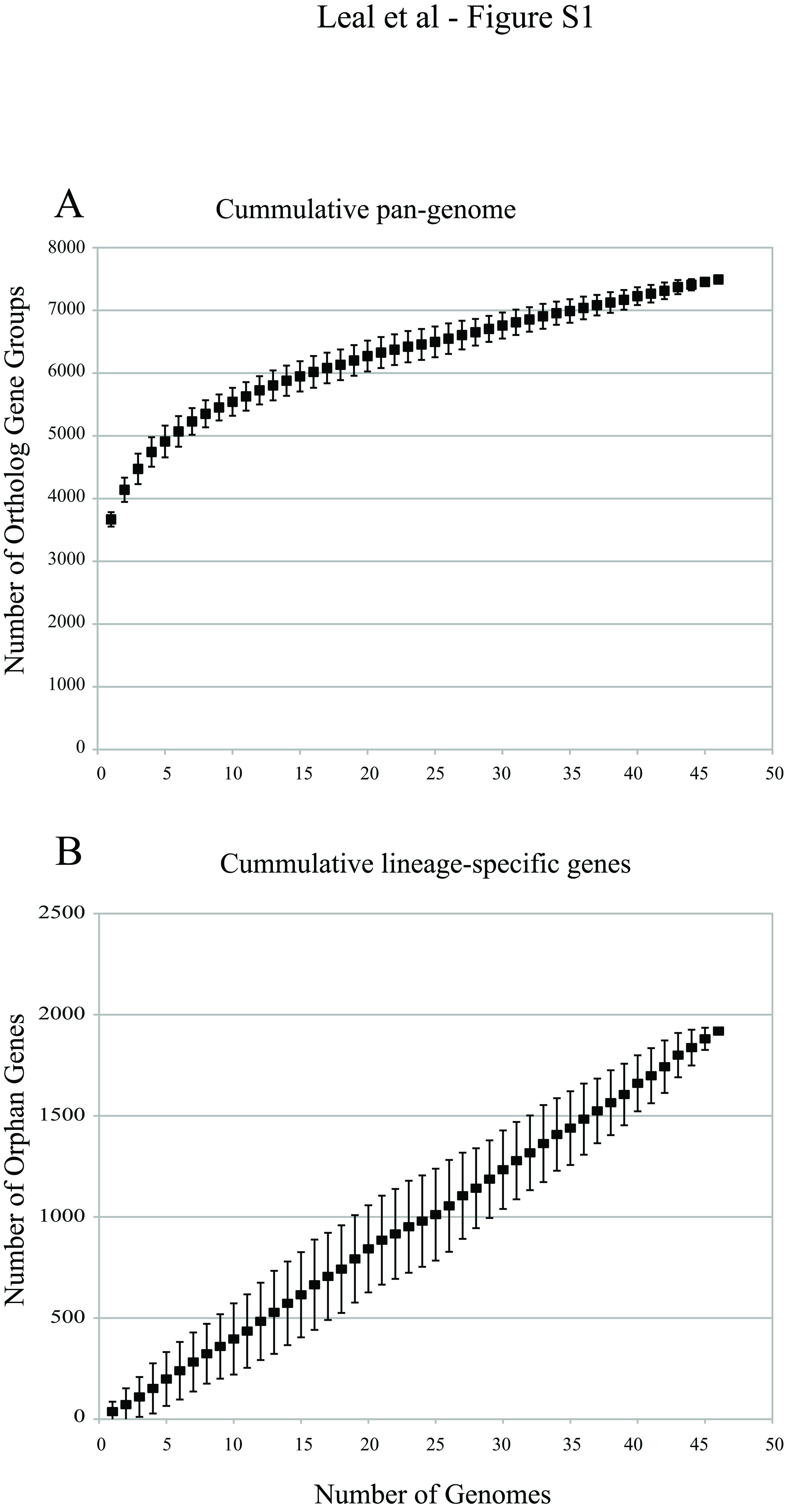

Supplement: FIGURE S1 — Evaluation of overall gene content from the genomes sequenced in this study. (A) Cumulative curve of Acinetobacter baumannii pan-genome using as proxy the number of new ortholog group of genes detected by ORTHOMCL software with 100 replicates. (B) Cumulative graph showing the number of unique genes after the addition of each new strain, 1,000 replicates. [file Image_1.jpg]

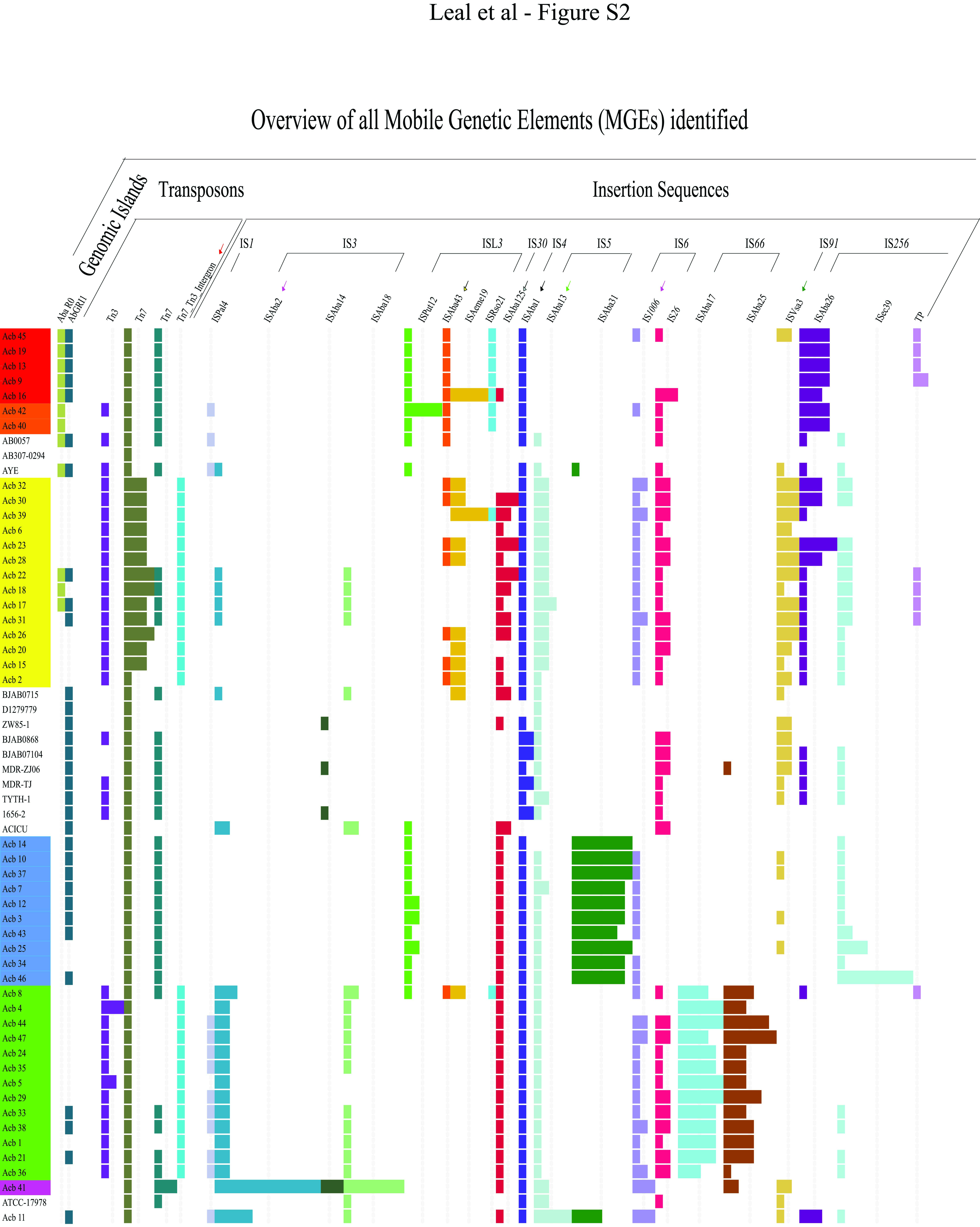

Supplement: FIGURE S2 — Full list of mobile genetic elements (MGEs) characterized by the homology search with ISFinder database are displayed in the right side of the figure and including genomic islands, transposons and insertion sequences (IS), the latter sorted according to the IS family to which they belong to. Since the MGEs can be found in multiple copies in each genome, the width of the colored rectangles reflects the number of copies found for each. The STs follow the colors displayed in Figure 1. [file Image_2.jpg]

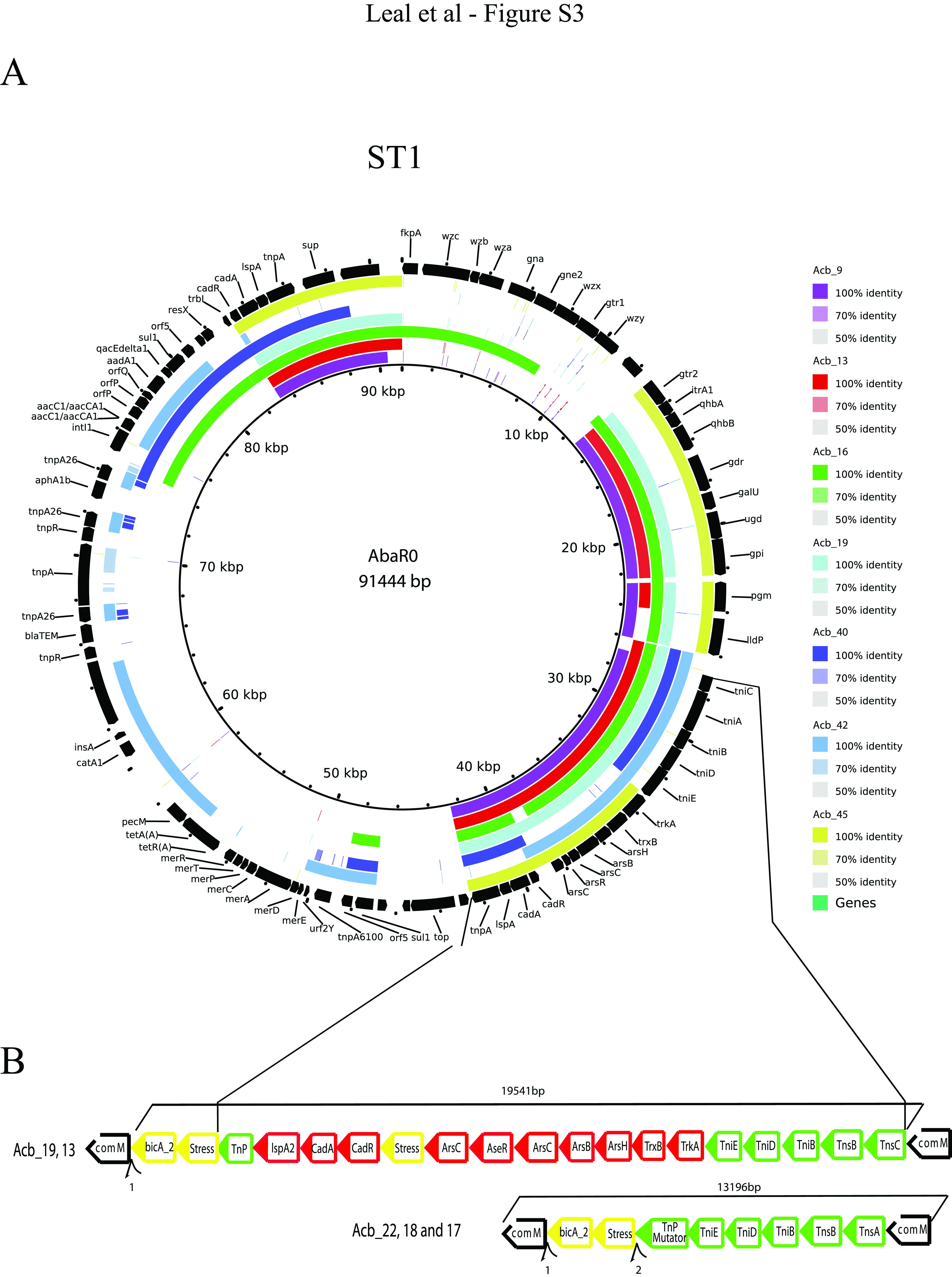

Supplement: FIGURE S3 — Regions from AbaR0 genomic islands found in the five ST1 and two ST881 strains. (A) Circular plot with regions with high identity from pair wise Blast comparisons between a reference AbaR0 island first detected in a A. baumannii strain from Australia (Hamidian et al., 2014) and the contigs derived from the sequenced Recife strains. (B) Striking examples of the AbaR0-like island dynamics found in a single contig flanked by the comM gene. A schematic representation of the AbaR0 island backbone transposons found in Acb_19 and Acb_13 (both ST1 strains) as well as Acb_22, Acb_18, and Acb_17 (all from ST79) is shown. Green boxes are MGEs related genes, red boxes define the resistance related genes, and yellow boxes represent the genes found in the islands sequenced in this study, but which are absent from the original AbaR0. It is important to note that, unless stated, this representation of the segments homologous to the AbaR0 and AbGRI1 islands found in the sequenced strains should not be interpreted as contiguous contigs. Only the presence or absence of genes originally found in an island should be considered. Any new gene present only in the newly sequenced strains will not be depicted in this figure. [file Image_3.jpg]

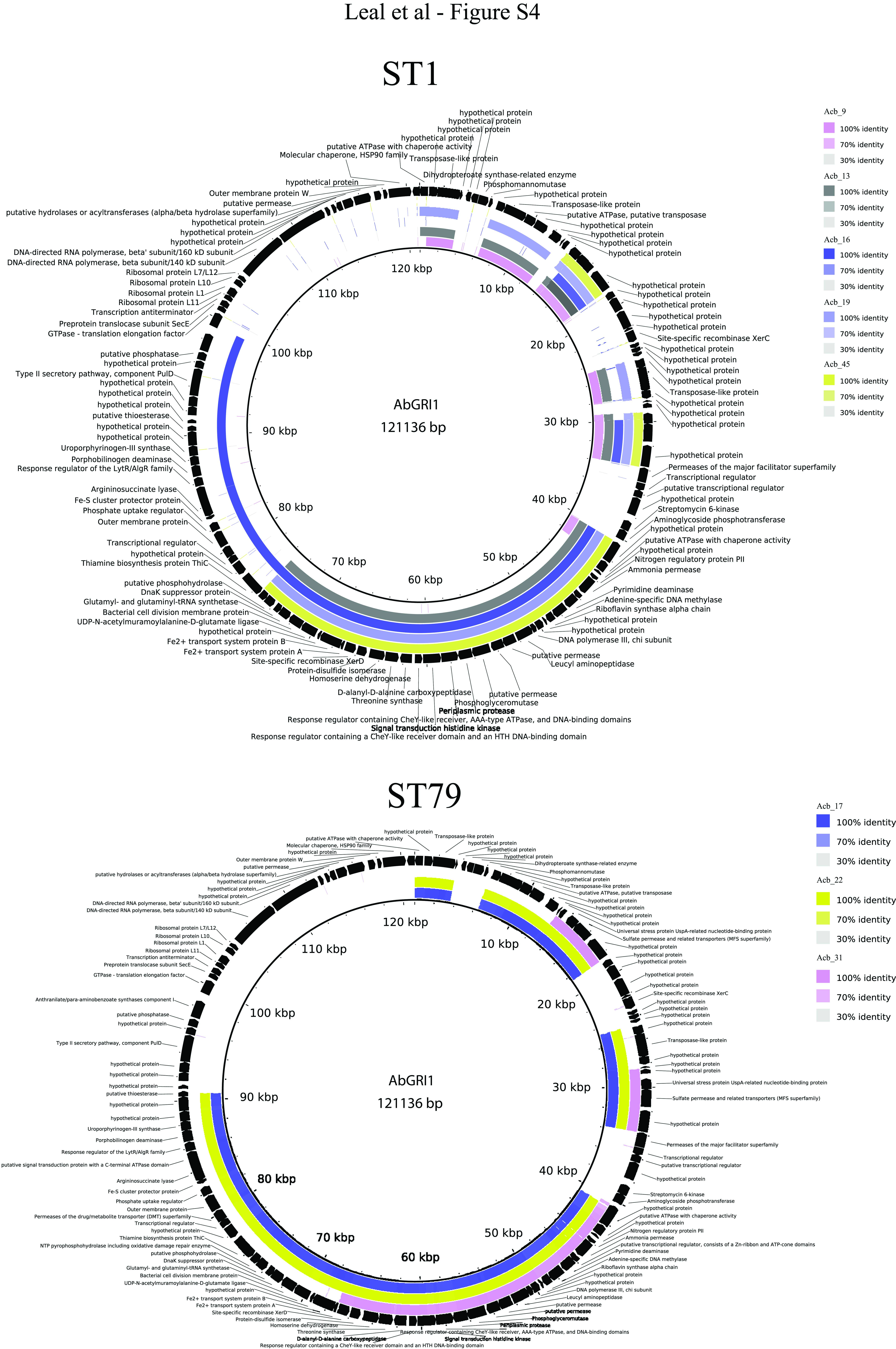

Supplement: FIGURE S4 — Regions from AbGRI1 genomic islands found in ST1 and ST79 strains. Only the presence or absence of genes originally found in an island should be considered. Any new gene present only in the newly sequenced strains will not be depicted in this figure. [file Image_4.jpg]

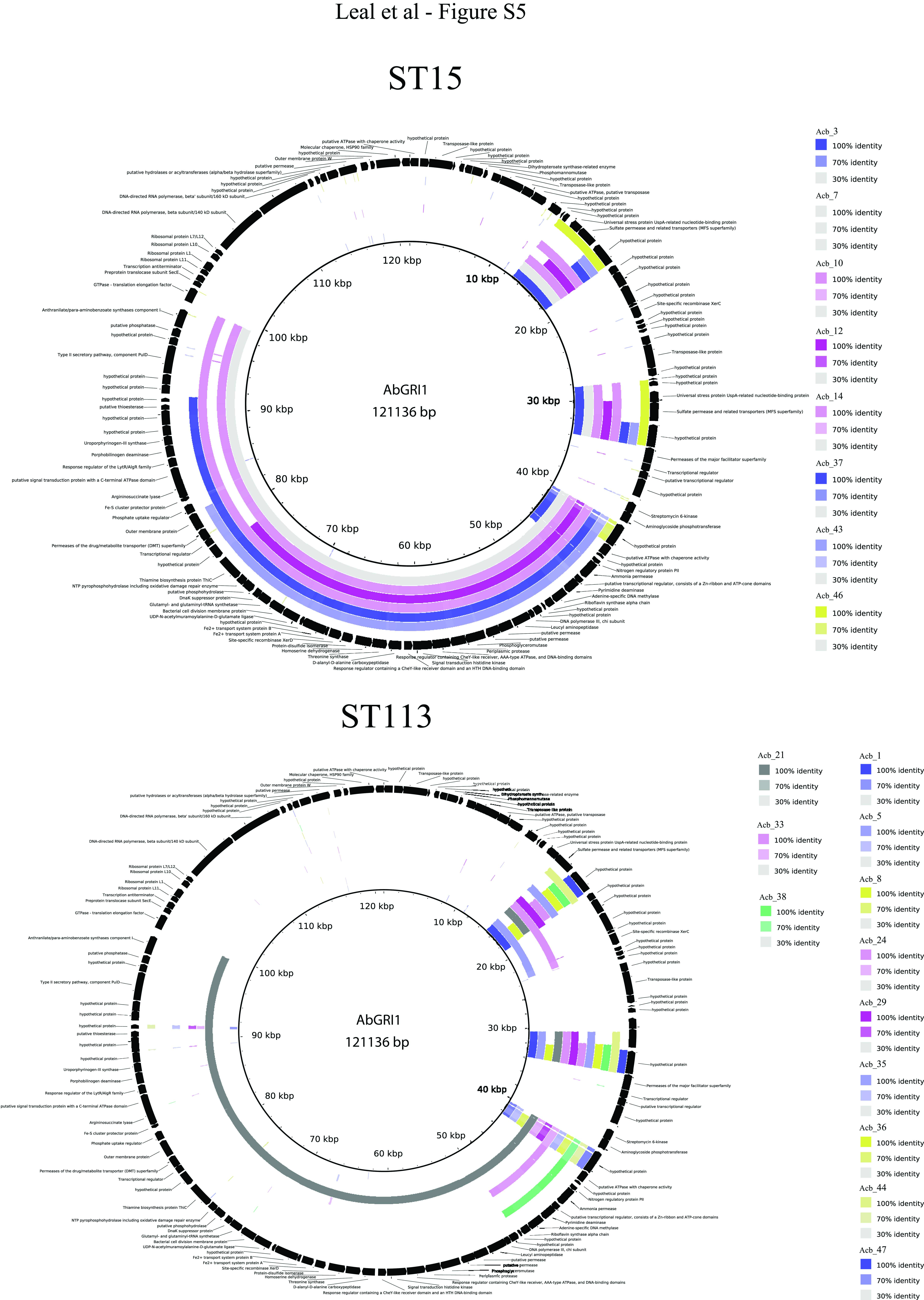

Supplement: FIGURE S5 — Regions from AbGRI1 genomic islands homologs found in ST113 and ST15 strains. Only the presence or absence of genes originally found in an island should be considered. Any new gene present only in the newly sequenced strains will not be depicted in this figure. Based on this analysis, for the ST113 strains, only Acb_21, Acb_33, and Acb_38 strains were considered to have true segments of an AbGRI1 island. [file Image_5.jpg]

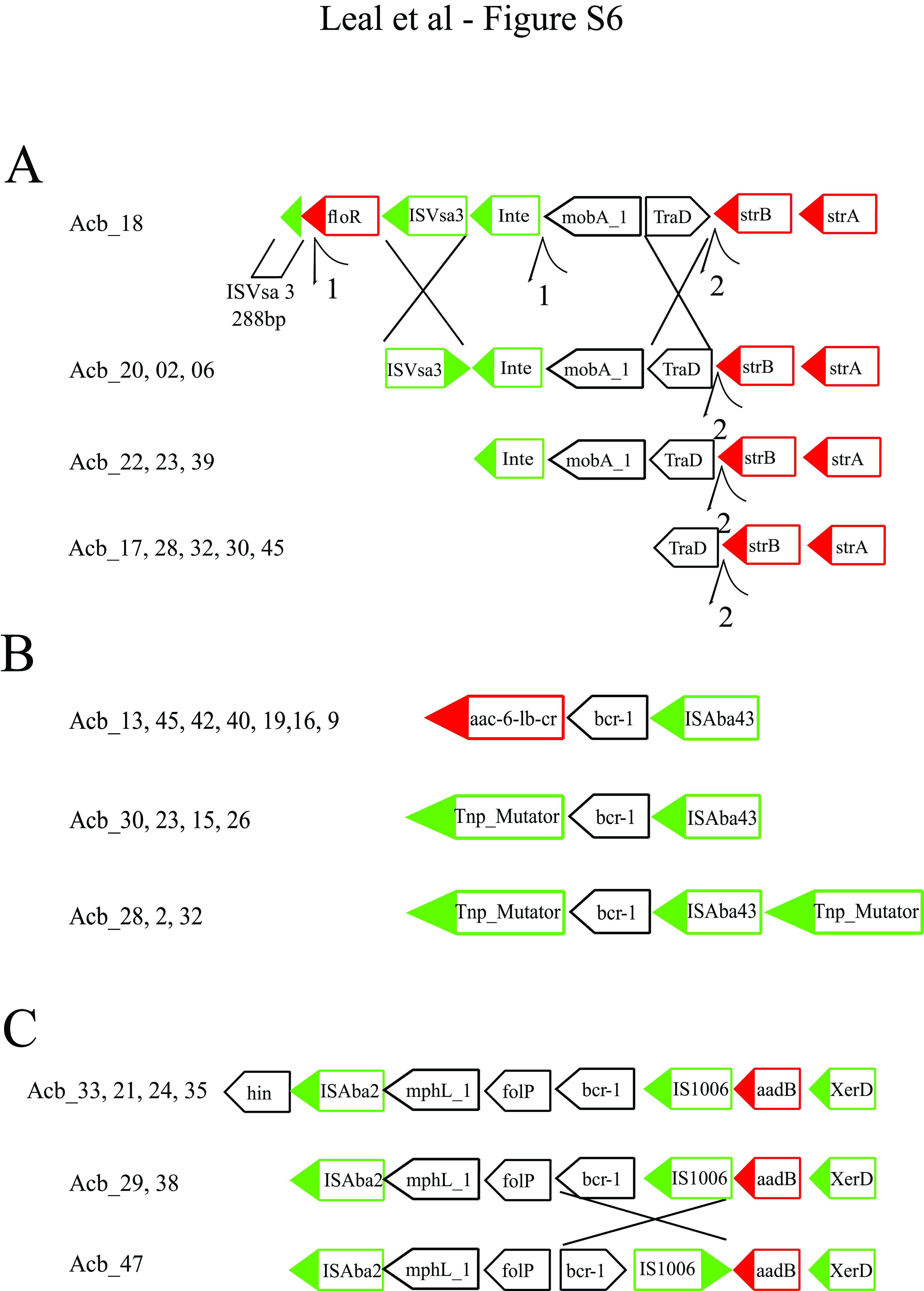

Supplement: FIGURE S6 — Other resistance and virulence genes found associated (in the same contigs) with transposition related genes and their assembly variability among the strains sequenced in this study. (A) Variations associated with the streptomycin (strB and strA) resistance genes, linked or not to ISVsa3 and transposable elements. (B) Variations associated with the bcr-1 gene and the ISAba43 insertion. (C) Alternative profiles for the elements flanking the aadB resistance gene. [file Image_6.jpg]
